# Refining Zooplankton Diet Composition Studies Over Short and Long Time Scales by Combining 18S Metabarcoding With Fatty Acid Analyses

**DOI:** 10.1111/1755-0998.70030

**Published:** 2025-08-18

**Authors:** Nora‐Charlotte Pauli, Katja Metfies, Stefan Neuhaus, Martin Graeve, Alison C. Cleary, Morten H. Iversen, Bettina Meyer

**Affiliations:** ^1^ Alfred Wegener Institute Helmholtz Centre for Polar and Marine Research Bremerhaven Germany; ^2^ Institute for Chemistry and Biology of the Marine Environment (ICBM), Carl‐ von‐ Ossietzky University of Oldenburg Oldenburg Germany; ^3^ Helmholtz Institute for Functional Marine Biodiversity (HIFMB) Oldenburg Germany; ^4^ Department of Natural Sciences University of Agder Kristiansand Norway; ^5^ British Antarctic Survey Cambridge UK; ^6^ MARUM and University of Bremen Bremen Germany

**Keywords:** diet composition, fatty acids, krill, metabarcoding, salps, zooplankton

## Abstract

Understanding diet composition is essential for unravelling trophic interactions in aquatic ecosystems. DNA metabarcoding, utilising various variable regions of the 18S rRNA gene, is increasingly employed to investigate zooplankton diet composition. However, accurate results depend on rapid inactivation of digestive enzymes and DNA nucleases through proper sample processing and preservation. In this study, we compare the prey communities of Antarctic krill retrieved from the 18S variable regions V4 and V7 and assess how different processing treatments affect the detected prey composition of both krill and salps. Our findings highlight the critical importance of prompt sample processing for species with highly efficient digestive enzymes, such as krill, to preserve rapidly digested prey, including gelatinous plankton. Comparative analyses of the V4 and V7 regions revealed significantly different prey communities within the same krill samples, indicating that these regions may not be suitable for direct comparisons within or across studies. To complement molecular approaches, we also analyse fatty acids (FA) as trophic markers which provide insights into dietary habits over both short and long time scales. By comparing FA signals from stomach and tissue samples of the same krill and salp individuals, we identified significant differences in trophic markers representing different plankton groups. These findings emphasise the necessity of separating digestive tract from tissue to distinguish between short‐ and long‐term diet signals. Furthermore, integrating FA analysis with metabarcoding offers valuable insights into zooplankton digestion efficiency across taxonomic levels. This combined approach enhances our understanding of zooplankton feeding ecology and trophic interactions in marine ecosystems.

## 1 Introduction

Understanding zooplankton diet composition is critical to unravelling food web interactions, ecosystem functioning and species‐specific feeding preferences (Pompanon et al. 2012; Troedsson et al. 2009). Traditional methods, such as microscopy, are time‐ and labour intensive, require taxonomic expertise and are biased against soft‐bodied prey like gelatinous plankton (Pompanon et al. 2012; van der Loos and Nijland 2021). Stomach content analyses typically provide short‐term dietary insights into recently consumed prey, while biomarkers like fatty acids (FA) and stable isotopes reflect long‐term trophic patterns but with lower taxonomic resolution (Dalsgaard et al. 2003). Combining these approaches, we can deepen our understanding of zooplankton feeding ecology.

FA are widely used to study trophic relationships, including for key Antarctic zooplankton species, such as krill, salps and copepods across various regions, life stages and seasons (Graeve, Hagen, and Kattner 1994; Meyer et al. 2002; Schmidt et al. 2006; Stübing et al. 2003; von Harbou et al. 2011). Most studies use whole animals, often pooling several individuals of one species to account for low biomass (Auel et al. 2002; Hiltunen et al. 2015). However, information regarding whether the digestive tract was removed before the extraction of FA is often missing (Ju and Harvey 2004; Reiss et al. 2015; Stübing et al. 2003). Using whole animals complicates the differentiation between short‐term and long‐term FA signals, as FA from prey in stomach contents are mixed with those in predator tissue. Separating the digestive tract, however, enables clearer differentiation between short‐ and long‐term dietary signals, though this practice remains infrequent (Cripps and Hill 1998; Schmidt et al. 2006).

DNA metabarcoding, i.e., massive parallel sequencing of DNA bulk samples using universal markers (Bucklin et al. 2016; Pompanon et al. 2012), was introduced as a tool for zooplankton diet studies to overcome some of the limitations in traditional approaches, including low taxonomic resolution and high expenditure of time and effort (de Sousa et al. 2019; Metfies et al. 2014; Pompanon et al. 2012). One of the most frequently used genetic markers for barcoding is the gene of the small subunit ribosomal RNA (18S rRNA), a multi‐copy gene containing both conserved and variable regions (Bucklin et al. 2016; Mallatt et al. 2004). Within the 18S rRNA, different variable regions have been used to study plankton biodiversity and community structure, including variable regions V4 (Fadeev et al. 2018; Metfies et al. 2014), V7 (Gast et al. 2004), V9 (Amaral‐Zettler et al. 2009) and the combined regions V7–V9 (Hirai et al. 2015).

In diet studies of marine metazoans, including fish, krill and salps, variable regions V4 and V9 have been shown to allow for direct comparison of diets between different species (Albaina et al. 2016; Metfies et al. 2014; Pauli et al. 2021). For Antarctic krill, a key species in the Southern Ocean ecosystem, the published diet studies using 18S metabarcoding were based on variable regions V7 and V4 (Cleary et al. 2018; Pauli et al. 2021). However, to what extent these variable regions of the 18S gene differ and/or are comparable remains unclear. Studies comparing regions V4 and V9 suggested that region V4 resembles the full‐length SSU gene more accurately than region V9 (Dunthorn et al. 2012). Another study found that region V9 might detect a broader range of taxa, while region V4 facilitates the distinction between closely related taxa (Stoeck et al. 2010). However, to our knowledge, no study has directly compared regions V4 and V7, particularly in the context of diet composition.

In addition to choosing a suitable genetic marker, successful metabarcoding depends on various factors, including a rapid deactivation of digestive enzymes and DNA nucleases (Bucklin et al. 2016; van der Loos and Nijland 2021). Thus, a suitable preservation method is crucial for successful extraction and amplification of DNA (Strauss 1998). Freezing and ethanol are the most common preservation methods in diet studies (Albaina et al. 2016; Cleary et al. 2018; Metfies et al. 2014). Both of these preservation methods are often a compromise between rapid fixation to preserve DNA and collecting additional parameters, such as size, stage and sex of the dissected animals needed to bring diet studies into a broader context. Previously, Passmore et al. (2006) reported challenges in amplifying DNA extracted from krill stomachs preserved at −80°C as samples thawed during dissection and showed improved results when preserving krill in ethanol. However, it remains unclear how the different pre‐processing treatments before and after preservation affect the prey composition in metabarcoding libraries.

This study refines zooplankton diet analyses by combining 18S metabarcoding and FA approaches to capture dietary signals across temporal scales. Using two key species in the Southern Ocean ecosystem, Antarctic krill ( *Euphausia superba* ) and the pelagic salp species *Salpa thompsoni* , we address the most critical methodological challenges outlined above to guide future experimental design for studies of zooplankton diet over short‐ and long‐term time scales:

- Comparing the stomach content of krill, determined by variable regions V4 and V7, addresses the explanatory power of different molecular markers.
- The impact of pre‐processing treatments on metabarcoding libraries, addressed by comparing two treatments using krill and salp stomachs.
- The effect of separating stomach and tissue on FA trophic markers in krill and salps.
- The synergistic value of integrating 18S metabarcoding (short‐term) with FA analysis (long‐term).

## 2 Materials and Methods

### 2.1 Field Sampling

Antarctic krill ( *Euphausia superba* Dana, 1850; hereafter referred to as krill) and the salp species *Salpa thompsoni* Foxton, 1961 (hereafter referred to as salps) were collected during the PS112 research cruise aboard RV Polarstern between March and May 2018 along the Antarctic Peninsula (Table S1). Sampling was performed using oblique net hauls with Isaacs‐Kidd Midwater Trawls (IKMT, 505 μm mesh size) and Rectangular Midwater Trawls (RMT 8 + 1 m^2^; with mesh sizes of 2 cm and 320 μm, respectively). The hauls targeted the upper 200 m of the water column and were conducted for 25 min at an average ship speed of 2 knots.

### 2.2 Sample Pre‐Processing Treatments and Preservation

Krill and salps were processed following standard protocol, which involved measuring length, determining sex, staging and freezing individuals at −80°C within 10 min of catch retrieval. Samples treated according to this protocol were used for both FA and molecular analyses and are hereafter referred to as samples with ‘delayed freezing’. Additionally, a subset of krill and salps were immediately frozen in liquid nitrogen and stored at −80°C within 5 min of retrieval to assess the impact of the delayed processing on the molecular analyses of the 18S variable region V4 (see Table 1 for an overview of all analyses, and Table S1 for sampling details).

**TABLE 1:** Overview of all performed analyses with details on species, treatment and sample size.

Species	Freezing	18S region	Stomach/Tissue	Sample size (*N*)
18S rDNA V4 vs. V7				
Krill	Delayed	V4	Stomach	19^a^
		V7	Stomach	21^a^
18S rDNA V4				
Krill	Delayed	V4	Stomach	10^b^
	Immediate	V4	Stomach	10^b^
Salps	Delayed	V4	Stomach	10^c^
	Immediate	V4	Stomach	10^c^
FA				
Krill	Delayed	—	Stomach + Tissue	7
Salps	Delayed	—	Stomach + Tissue	8

*Note:* Treatments (before conducting molecular and chemical analyses) included delayed and immediate freezing. For fatty acid analyses, stomach and tissue samples from the same individuals (krill and salps) were analysed separately.

^a^ Collected at five stations with 3–6 individuals per variable region each.

^b^ Collected at two adjacent stations in the Weddell Sea.

^c^ Collected at one station around Deception Island. For more details on sampling region, date and time, see Table S1.

Freezing was chosen over ethanol preservation during the research cruise to ensure compatibility with water column samples collected simultaneously for plankton community analyses, which were filtered and stored at −80°C as standard practice (Pauli et al. 2021). In the home laboratory, krill and salp stomachs were dissected on ice to minimise thawing. Stomach samples for molecular analyses were kept frozen until DNA extractions. For FA analyses, stomachs were separated from the bodies and samples were lyophilised before being stored dry until lipid extraction.

### 2.3 Molecular Analyses

#### 2.3.1 DNA Extraction

Genomic DNA was extracted from krill and salp stomach samples using the NucleoSpin Plant II Mini Kit (Macherey‐Nagel, Germany) according to the manufacturer's protocol. Stomach samples were kept frozen until lysis buffer was added to preserve DNA integrity. Subsequently, samples were carefully homogenised with a pestle to ensure thorough cell disruption. DNA was eluted in two steps, each using 30 μL of elution buffer, to maximise recovery. The concentration of the extracted DNA was quantified using a fluorescent DNA‐binding dye (QuantiFlour, Promega, USA).

#### 2.3.2 PCR Amplification

##### 2.3.2.1 Salp Stomachs

Variable region 4 (V4) of the 18S rRNA gene from salp stomachs was amplified using primers 528iF (5′‐GCGGTAATTCCAGCTCCAA‐3′) and 964iR (5′‐ACTTTCGTTCTTGATYRR‐3′), targeting a 436 bp long fragment (Fadeev et al. 2018). Previous studies investigating the gut content of the same salp species with similar primers (528F, 690R) reported salp‐derived reads constituting < 3% of total reads (Metfies et al. 2014). This indicates that the use of a salp‐specific blocking probe is unnecessary.

Polymerase chain reaction (PCR) was performed in two steps following the Illumina 16S Metagenomic Sequencing Library Preparation protocol (Part # 15044223 Rev. B). In a first PCR, we used 25 μL reaction mixture containing 2.5 μL DNA template (5 ng μL^−1^), 12.5 μL of KAPA HiFi HotStart ReadyMix PCR Kit (Kapa Biosystems), and 5 μL each of the forward and reverse primer (1 μM), each of which contained an overhang adapter. Amplification was conducted in 25 cycles under the following conditions: denaturation at 95°C for 30 s, annealing at 55°C for 30 s and elongation at 72°C for 30 s, followed by a final elongation step at 72°C for 5 min and subsequent cooling to 4°C. The amplicon from the first PCR was purified before Illumina adapters were attached to the overhang adapters in a second, short‐cycle PCR (8 cycles, identical temperature profile) using 5 μL amplicon, 5 μL of each of the Index primers, 25 μL KAPA PCR kit and 5 μL PCR grade water, followed by a second purification step. Each PCR batch included three negative controls without template DNA to monitor potential contaminations.

##### 2.3.2.2 Krill Stomachs

Variable region V4 of the 18S rRNA gene from krill stomachs was amplified using the same procedure described for salp stomachs, with an additional step to mitigate predator DNA amplification. Specifically, a blocking probe (5′‐GACGGGCTTTAGCGTTC‐3′, 5 μL of 19°μM probe; biomers.net) was included in the PCR reaction (Pauli et al. 2021). The blocking probe prevents amplification of krill's own DNA, which could originate from residual krill tissue in the stomach contents (e.g., remains from dissection, through feeding on moults or cannibalism) and potentially overshadow prey DNA sequences (Pauli et al. 2021; Vestheim and Jarman 2008). To address variability associated with low sequencing yields, krill V4 samples were sequenced in triplicates. This approach pooled information across different sequencing reactions, enhancing data readability and reducing biases arising from stochastic effects.

Variable region 7 (V7) of the 18S rRNA gene from krill stomach content samples was amplified following the protocol established by Cleary et al. (2018). This region has been previously utilised to investigate krill stomach content (Cleary et al. 2018, 2012), enabling comparisons with existing datasets. To date, these studies, along with a previous study from our lab using the V4 region (Pauli et al. 2021), represent the limited body of research applying 18S rRNA variable regions to analyse krill diets. Given this context, we opted to use the V7 region specifically for krill to allow a direct comparison with past studies, while not extending this approach to salps.

Amplification was performed using a two‐step PCR approach. The first PCR amplified a 240 bp fragment using primers modified from Gast et al. (2004); 960F (eukaryotic, 5′‐GGC TYA ATT TGA CTC AAC RCG‐3′; 0.5 μM) and 1200R (universal, 5′‐GGG CAT CAC AGA CCT G‐3′; 0.5 μM). To block krill's own DNA, a peptide nucleic acid (PNA) probe (5′‐CGT CGG GTT GTC TTG‐3′; 20 μM) was included in the reaction. In the second PCR, primers containing Illumina adapters (0.2 μM each) were used to re‐amplify the product from the first PCR in nine cycles. Two negative controls (without DNA template) were included and carried through all PCR steps to monitor potential contamination. The two‐step design allowed the PNA probe to bind at a higher temperature than the primers in the initial PCR, which have a lower melting point. This temperature difference ensured that the PNA probe effectively outcompeted the primers, while the longer Illumina adaptor primers with higher melting points were utilised in the subsequent amplification step.

#### 2.3.3 Sequencing and Analysis Pipeline

Sequencing library preparation as outlined above was carried out using the amplified DNA products following the Illumina 16S Metagenomic Sequencing Library Preparation protocol (Part # 15044223 Rev. B). The final library pool was prepared with 20% PhiX control and adjusted to a final DNA concentration of 12 pM for the V7 samples and 14 pM for the V4 samples.

Paired‐end sequencing was conducted on an Illumina MiSeq platform across four separate runs, generating 2 × 300 bp sequences. To accommodate the large number of samples, triplicates of the V4 libraries for krill were sequenced in two distinct runs, while krill stomach content samples using region V7 and salp stomach content samples were sequenced in individual runs. Sequence analyses were performed in R, v.3.6.1 (R Core Team 2024) using a modified version of the Divisive Amplicon Denoising Algorithm (DADA2) pipeline tutorial (https://benjjneb.github.io/dada2/tutorial.html), with the DADA2 package v.1.14.1 (for details see [Link], [Link], [Link], [Link], [Link], [Link]; Callahan et al. 2016). Taxonomy was assigned using the PR2 database, v.4.12.0 (Guillou et al. 2012).

Following taxonomic assignment, predator DNA was removed from each of the datasets using the *phyloseq* package in R, v.1.28.0 (McMurdie and Holmes 2013). This process excluded all ‘Malacostraca’ sequences, including genera *Euphausia*, *Meganyctiphanes* and *Thysanopoda* from krill samples, as well as all Tunicata sequences, including *Salpa thompsoni* and *Salpa* sp. from salp samples. Additionally, sequences that could not be assigned to a taxonomic level lower than ‘Phylum’ (< 250 reads in total) were aligned to a reference database using the *blastn* tool (Camacho et al. 2009) and excluded if no taxonomic match (> 98% identity) was found. Single sequences of non‐marine taxa, possibly resulting from contamination during laboratory procedures, were also excluded.

### 2.4 Fatty Acid Extractions

Fatty acids (FA) were extracted from dissected, lyophilised and homogenised krill and salp specimens excluding the stomachs, treated according to standard protocol with delayed freezing (i.e., krill and salps were measured before freezing). Whole, individual krill were used, with the head and large chitin parts removed (*n* = 7). For salps, multiple individuals of the same size and developmental stage were pooled (*n* = 17 pooled to *n* = 8; Table S1) to compensate for their high water content and low organic material (Dubischar et al. 2011). Additionally, FA were extracted from the lyophilised and homogenised stomachs of the corresponding gutted individuals using the same protocol.

Lipid extraction was performed using the method outlined by Kattner and Fricke (1986). Extraction was performed with 2:1 v:v dichloromethane/methanol, followed by transesterification with 3% sulphuric acid in methanol (4 h at 80°C). Fatty acid methyl esters and fatty alcohols were then extracted with cyclohexane. Analysis was conducted on a gas chromatograph (GC 6890N, Agilent) equipped with a split/split‐less injector (250°C) and flame ionisation detector (FID) at 280°C. Two chromatographic systems were employed depending on the final concentration of the extracted compounds. First, a DB‐FFAP column (30 m, 0.25 mm diameter, 0.25 μm film thickness) was used with a temperature program at 4°C min^−1^ from 160°C to 240°C, and a subsequent hold for 15 min at the final temperature. Second, a DB‐FFAP column (60 m) with identical dimensions was employed with temperatures from 80°C to 240°C (1st ramp 80°C to 160°C with 20°C min^−1^, 2nd ramp from 160°C to 240°C with 2°C min^−1^ and a final 20 min hold). FA and fatty alcohols were identified using known commercial and laboratory standards.

### 2.5 Statistical Analyses

#### 2.5.1 Metabarcoding Libraries

Sequencing data were analysed in R, v.4.3.2 (R Core Team 2024), treating all data as compositional (Gloor et al. 2017). Amplicon sequence variants (ASV) with a relative abundance below 0.01% across all samples were excluded prior to performing a centred‐log‐ratio (clr) transformation. This log transformation was based on the geometric mean of ratio‐transformed data, using the *codaSeq.clr* function in *CodaSeq* (Gloor and Reid 2016). To assess differences in diet communities, principal component analysis (PCA) and permutational multivariate analyses of variance (PERMANOVA) were employed. Prior to these analyses, homogeneity of group dispersion was checked using *betadisper*, followed by analysis of variance (ANOVA) in the *vegan* package (Oksanen et al. 2024).

Krill V7 libraries were predominantly composed of sequences from a eugregarine parasite, potentially obscuring other sequences. To facilitate a meaningful comparison between the V4 and V7 libraries, these parasite sequences were removed, and both libraries were rarefied to an even sampling depth using the *rarefy_even_depth* function in *phyloseq* (McMurdie and Holmes 2013). Samples with fewer than 200 reads were excluded, resulting in *n* = 16 for V4 and *n* = 6 for V7. A fixed seed was used to ensure reproducibility during random subsampling. Other parasite sequences, e.g., Syndiniales, were retained for subsequent analyses. Data handling and visualisations were conducted using *FactoMineR* (Lê et al. 2008), *ggplot2* (Wickham 2016), and *tidyverse* (Wickham et al. 2019). Figures were further refined (e.g., font size adjustment) in Inkscape v.1.3. Raw sequences and metadata have been deposited in the European Nucleotide Archive (ENA) under accession number PRJEB43900, via the data brokerage service of the German Federation for Biological Data (GFBio; Diepenbroek et al. 2014), adhering to the Minimal Information about any (X) Sequence (MIxS) standard (Yilmaz et al. 2011).

#### 2.5.2 Fatty Acids

Dietary FA markers for the three main plankton groups are established in the literature: diatoms, dinoflagellates and calanoid copepods (Dalsgaard et al. 2003; Graeve, Hagen, and Kattner 1994; Graeve, Kattner, and Hagen 1994). Accordingly, we used FA 16:1(*n*−7) and 20:5(*n*−3) as markers for diatoms, 18:4(*n*−3) and 22:6(*n*−3) for dinoflagellates and 20:1(*n*−11/*n*−9/*n*−7) and 22:1(*n*−11/*n*−9/*n*−7) for calanoid copepods.

In addition to these traditional FA marker combinations for diatoms and dinoflagellates, we applied two alternative combinations that excluded FA 20:5(*n*−3) and 22:6(*n*−3). These FAs are major membrane components and are largely unaffected by dietary changes (Lee et al. 2006). Consequently, marker combinations including these FAs, particularly from tissue samples, may overestimate the contribution of diatoms and dinoflagellates to zooplankton diet. As an alternative, we used 16:1(*n*−7) and all identified C16 polyunsaturated fatty acids (PUFAs; 16:2(*n*−4), 16:3(*n*−4), 16:4(*n*−1)) as markers for diatoms. For dinoflagellates, we included 18:4(*n*−3) and all C18 PUFAs (18:3(*n*−3), 18:2(*n*−3)) as additional marker combinations.

Differences in dietary markers between tissue and stomach samples of krill and salps were tested using Student's *t* test after confirming the assumptions of homogeneity of variances and normality of residuals in R, v.3.6.1 (R Core Team 2024). If these assumptions were not met, a Wilcoxon rank‐sum test was applied. Multivariate analyses of FA composition were conducted separately for krill and salps using weighted log‐ratio analysis in the *easyCODA* package, v.0.34.3 (Greenacre 2018).

## 3 Results

In this study, we analysed three datasets to address critical methodological challenges in diet composition studies.

- Metabarcoding of krill stomach content comparing variable regions V4 and V7.
- Metabarcoding of krill and salp stomach contents based on variable region V4, assessing the effects of two different processing treatments.
- Comparing long‐term fatty acid markers from tissue samples to short‐term markers from stomach samples, analysed in the same krill and salp individuals.

### 3.1 Metabarcoding of Krill Stomach Content Using 18S Variable Regions V4 and V7

To compare the performance of two molecular markers, 18S variable regions V4 and V7, we analysed krill stomach content collected from five stations along the Antarctic Peninsula (Table S1). A previous study conducted during the same cruise found no significant differences in krill diet composition between nearby stations within the same region (Pauli et al. 2021), allowing for the comparison of samples across adjacent stations.

Despite using a blocking probe for both markers, predator sequences (i.e., krill) constituted a significant proportion of the raw sequencing data (~50% in V4 libraries, ~95% in V7 libraries), with considerable variation among individual samples (20%–85% in V4, 80%–> 95% in V7 libraries). Repeated amplification and sequencing of several V7 samples yielded consistent results regarding community composition and blocking probe efficiency. Importantly, removing predator sequences did not alter the relative abundances of the prey taxa across samples. Rarefaction curves indicated sufficient sequencing depth for most samples (i.e., the curves reach plateau; Figure S1). Thus, predator sequences were excluded (Table 2), resulting in an average of 3500 sequences per sample (3.4%), aligning with previous studies on MiSeq platforms (Jungbluth et al. 2021; Rennstam Rubbmark et al. 2019) and the resulting datasets were considered suitable for cautious comparisons between the two sequencing libraries (for raw reads per sample an assigend taxonomy see Appendix S3,S5).

**TABLE 2:** Number of reads and amplicon sequence variants (ASVs) before and after the removal of predator sequences (krill or salps, respectively) for each of the three analysed datasets.

	Samples	Raw data		Removing predator sequences		Removing rare ASVs
		Reads	ASVs	Reads	ASVs	ASVs analysed^a^
Krill V4 vs. V7	40	2,562,349	3200	165,038	362	128
Salp V4	20	2,809,854	923	391,170	709	237
Krill V4	20	974,812	1652	836,422	960	723

^a^ ASVs with a relative abundance of > 0.01% across all samples.

**FIGURE 1:**
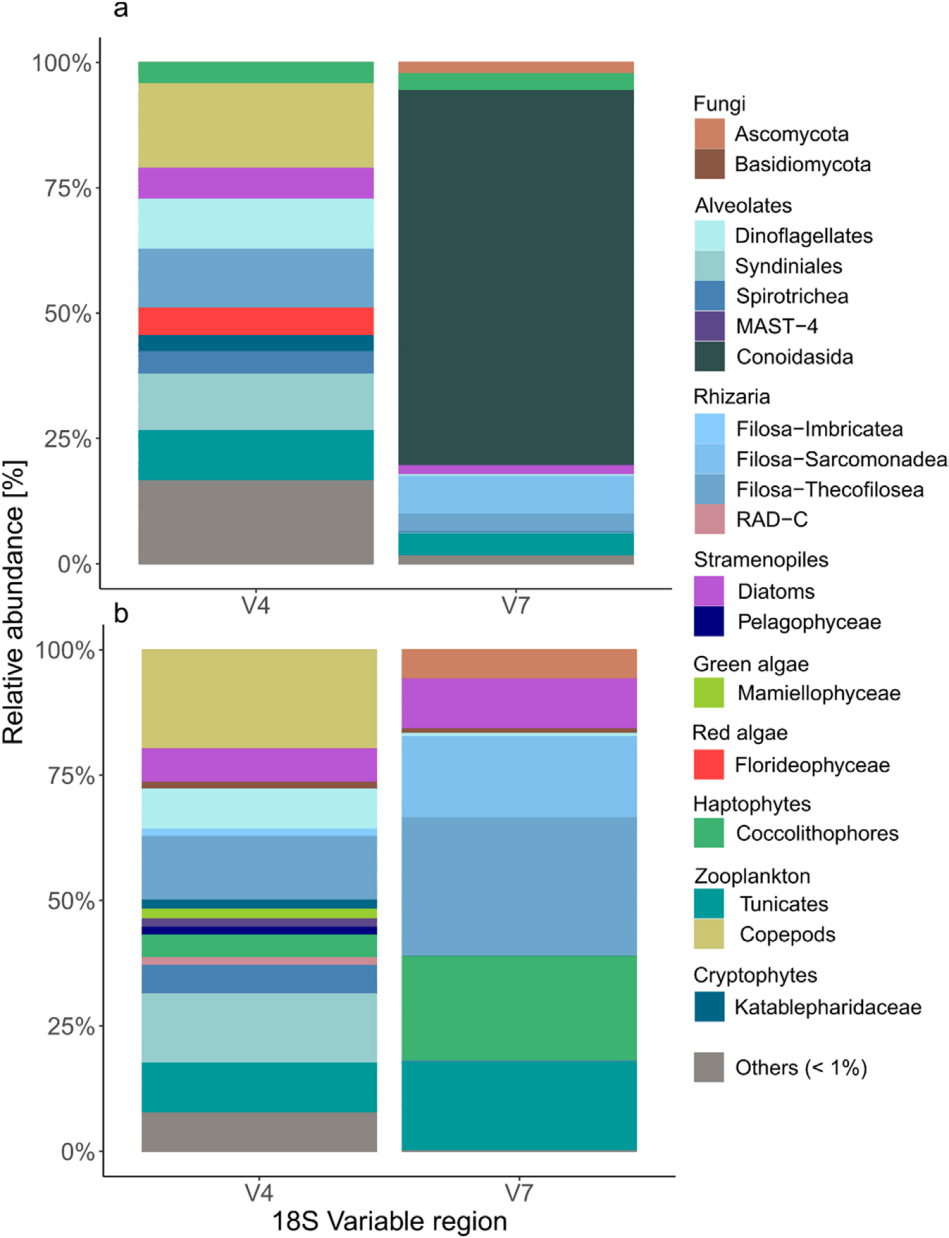
Relative abundance of taxa found in the stomach content of krill comparing 18S variable regions V4 and V7. (a) Relative abundance of taxa on the level of Class after removing predator DNA (krill's own DNA). (b) Dataset after the additional removal of sequences of the eugregarine parasite (Conoidasida, Alveolates) and rarefaction to an even sampling depth. Taxa with a relative abundance < 1% were grouped as ‘Others’.

After predator sequences were removed, V7 libraries were largely dominated by sequences of a eugregarine parasite (Conoidasida 74.9%, Figure 1a), which were absent from the V4 libraries. This parasite was identified as *Cephaloidophora* spp., a common endoparasite species of Antarctic krill (Takahashi et al. 2008). To enable better comparison between the two variable regions, parasite sequences were excluded, and both libraries were rarefied to an even sampling depth.

The rarefied datasets revealed clear differences in prey community composition between V4 (*n* = 16) and V7 (*n* = 6) libraries (Figure 1b). In V4 libraries, copepods (19.8%) and the parasitic dinoflagellate group Syndiniales (13.8%) were the most abundant taxa, but both were absent from V7 libraries. Other prominent taxa in V4 libraries included small flagellates (Filosa‐Thecofilosea 12.7%), salps (9.6%), dinoflagellates (7.9%) and diatoms (6.7%). In contrast, V7 libraries were dominated by small flagellates, specifically Filosa‐Thecofilosea (mainly *Ebria*, 27.6%) and Filosa‐Sarcomonadea (16.2%), the latter being absent from V4 libraries. C*occolithophores* (20.9%), salps (17.3%), and diatoms (9.9%) also showed higher abundance in V7 libraries compared to V4. Taxa with mean abundance below 1% were grouped as ‘Others’, accounting for 7.9% in V4 and 0.4% in V7 libraries.

**FIGURE 2:**
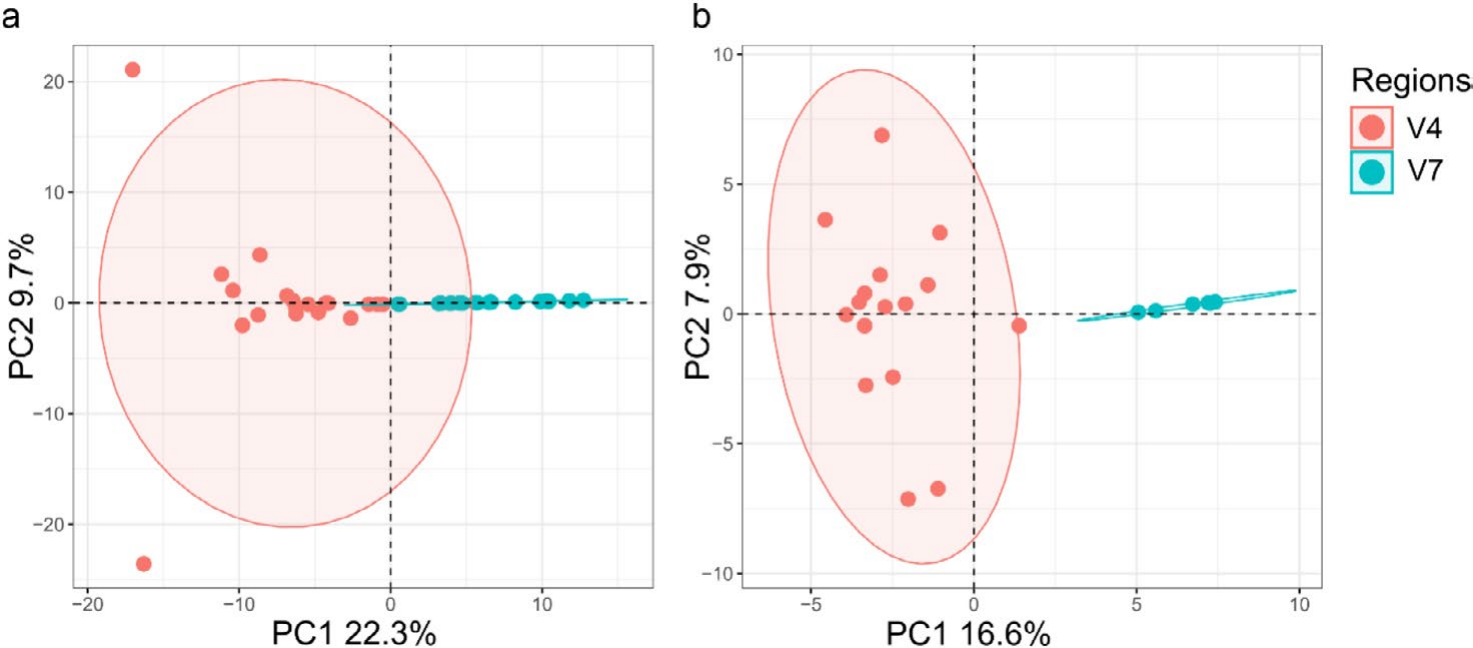
Principal component analysis of the centre‐log ratio transformed data (a) after the removal of predator (i.e., krill) sequences, and (b) after the additional removal of parasite sequences comparing V4 and V7 sequencing libraries. Ellipses were drawn with a 95% confidence interval. Plot was created using the FactoMineR package in R (Lê et al. 2008).

Principal component analysis (PCA) on log‐ratio transformed data without predator sequences revealed clustering of V4 and V7 samples along the first dimension (22% of explained variance; Figure 2a). V4 libraries exhibited a greater dispersion than V7 libraries (Figure S2), but a permutational multivariate analysis of variance (PERMANOVA), which is robust to dispersion effects, confirmed significant differences between the two groups (*p* > 0.001; Table S2). Removing eugregarine parasite sequences further enhanced the separation of V4 and V7 clusters, as evidenced by non‐overlapping 95% confidence ellipses in the PCA (Figure 2b; PERMANOVA *p* > 0.001, Table S2).

### 3.2 Effect of Different Pre‐Processing Treatments on 18S V4 Sequencing Libraries

#### 3.2.1 Krill Stomach Content

To evaluate the impact of two different processing treatments on the 18S sequencing libraries of krill stomach contents (variable region V4), we compared the following treatments: (a) krill with delayed freezing due to measurements and classification (*n* = 10; Table S1) and (b) krill immediately frozen at −80°C without prior handling (*n* = 10). All samples were collected from two adjacent stations in the Weddell Sea region (62°36–63°44 S, 54°34–56°30 W). After excluding predator sequences and rare sequences (< 0.01%), analysis was conducted on 723 ASVs (Table 2, for raw reads per sample and assigned taxonomy see Appendix S2,S3).

**FIGURE 3:**
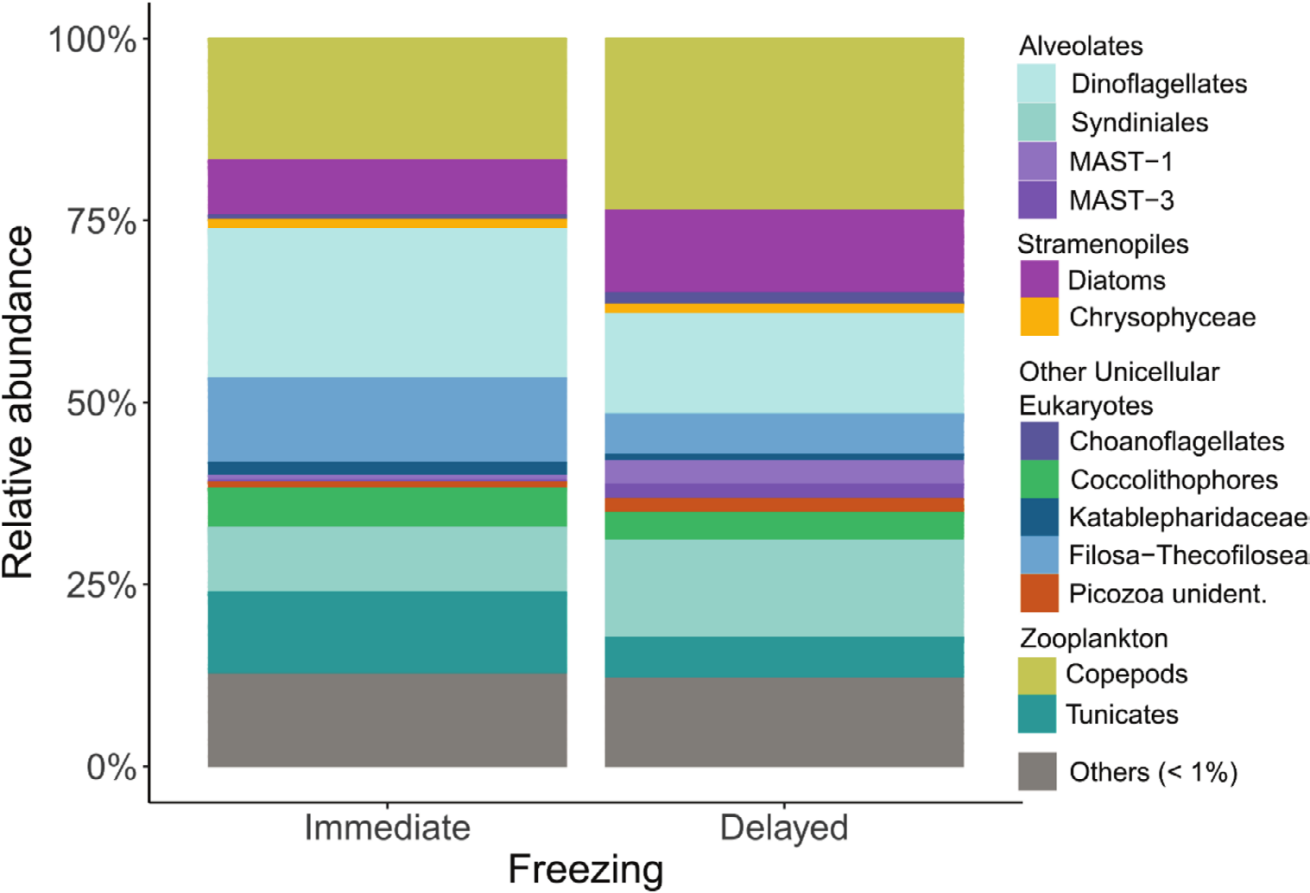
Krill stomach content. Relative abundance of 18S sequencing libraries of krill stomach content samples using variable region V4 comparing two pre‐processing treatments before molecular analyses were conducted. Samples that were frozen immediately after the catch was on board are compared to samples with delayed freezing due to measurements and staging before freezing krill at −80°C. Taxa with a relative abundance of < 1% were grouped as ‘Others’.

Both treatment groups exhibited a high proportion of copepod sequences, predominantly *Calanus* spp. and *Oithona* spp. However, copepod sequences were more abundant in samples with delayed freezing compared to immediately frozen samples (23.6% vs. 16.8%; Figure 3). Similarly, diatoms (11.4% vs. 7.5%) and the parasitic dinoflagellate group Syndiniales (13.4% vs. 8.9%) were more prevalent in samples with delayed freezing. In contrast, immediately frozen samples had higher proportions of dinoflagellates (20.4% vs. 13.8%), small flagellates (Filosa‐Thecofilosea, mainly *Ebria*; 11.6% vs. 5.6%) and salps (10.9% vs. 5.3%). Taxa contributing < 1% (mean across all samples) were categorised as ‘Others’, accounting for about 12% in both groups, with substantial variability among individual samples (Figure S3).

PCA revealed clustering of the two treatment groups along the first two dimensions, which explained 25.3% and 9.7% of the variance, respectively (Figure S4). A PERMANOVA confirmed significant differences between the groups (*p* = 0.009, Table S2). This result remained significant after excluding two potential outliers (*p* = 0.001, *F* = 1.31).

#### 3.2.2 Salp Stomach Content

For salps, we analysed the stomach contents using 18S metabarcoding of variable region V4, comparing salps that were immediately frozen (*n* = 10; Table S2) to those with delayed freezing (*n* = 10). All samples were collected at the same station near Deception Island (62°59.58′ S, 60°27.34′ W). After removing predator sequences and rare sequences (< 0.01%), analyses were conducted on 237 ASVs (Table 2, see Appendix S6,S7 for raw reads per sample and assigned taxonomy ).

**FIGURE 4:**
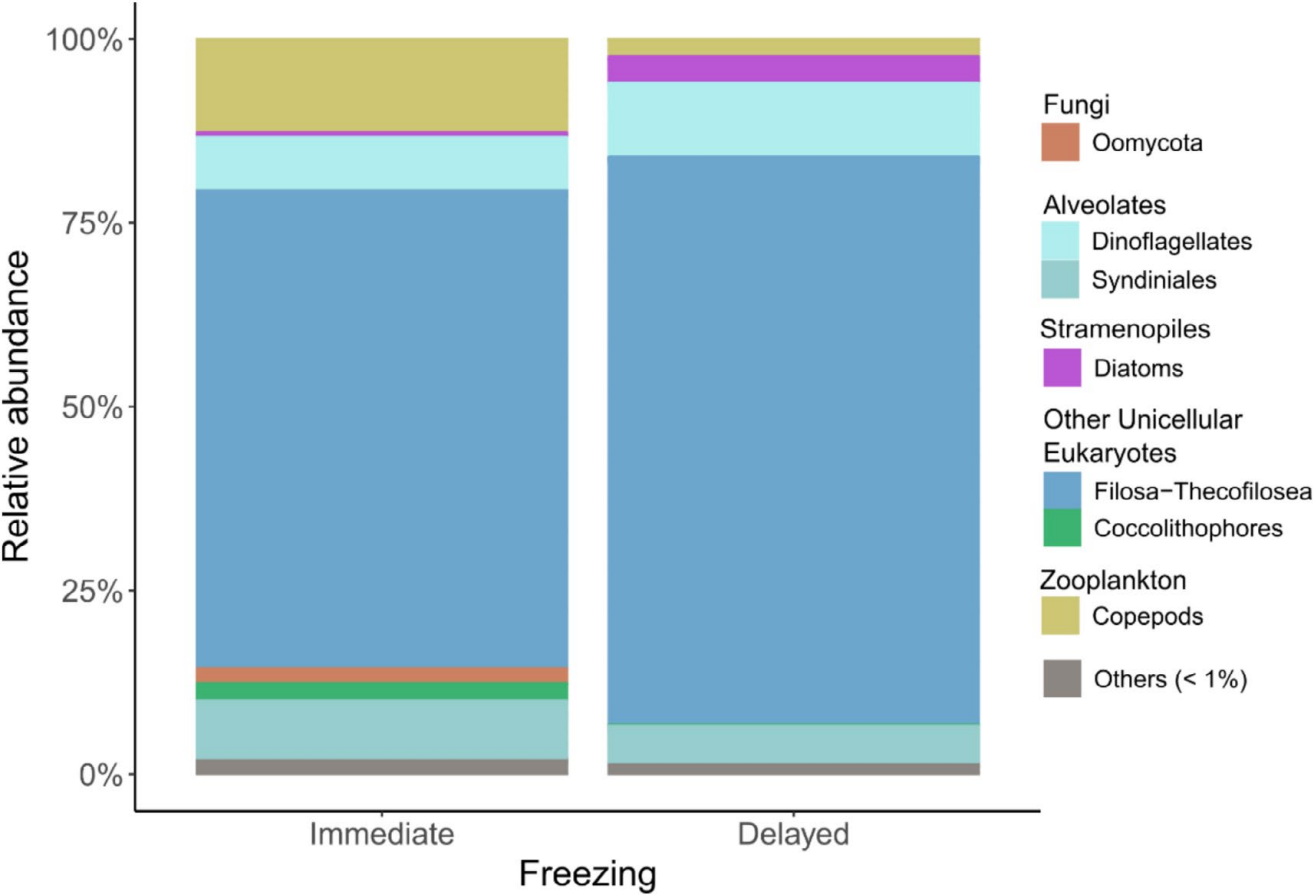
Salp stomach content. Relative abundance of 18S sequencing libraries of salp stomach content samples using variable region V4 comparing two pre‐processing treatments before molecular analyses were conducted. Samples that were frozen immediately after the catch was on board were compared to samples with delayed freezing due to measurements and staging before freezing salps at −80°C. Taxa with a relative abundance of < 1% were grouped as ‘Others’.

Both sequencing libraries were dominated by small flagellates (Filosa‐Thecofilosea), primarily *Ebria* spp. (Immediate 65% vs. Delayed 77.3%; Figure 4). *Ebria* spp. accounted for 40.2%–88.6% of sequences in individual salp stomachs, except for two stomachs in the immediately frozen samples, where this taxon was absent (Figure S5). In immediately frozen samples, copepods were the second most abundant taxon (12.8%), followed by the parasitic dinoflagellate group ‘Syndiniales’ (7.8%) and classic dinoflagellates (Dinophyceae, 7.3%). Conversely, copepods were far less abundant in samples with delayed freezing (2.3%), while dinoflagellates (10%) and diatoms (3.7%) were more prominent compared to immediate freezing. Taxa contributing < 1% (mean across all samples) accounted for 2.2% in immediately frozen and 1.6% in delayed frozen samples.

PCA explained 31.6% and 18.7% of the variance in the first two dimensions, respectively, but did not reveal distinct clusters of immediate or delayed frozen samples (Figure S6). Consistently, a PERMANOVA showed no significant differences between the two groups (*p* = 0.13, Table S2).

### 3.3 Fatty Acids

To assess the difference between short‐ and long‐term dietary signals, we analysed the fatty acid (FA) composition of stomach and tissue samples from the same krill and salp individuals. In total, 30 different FAs were identified (Table S3). Salps exhibited a higher proportion of polyunsaturated fatty acids (PUFA) in their stomachs (27.2% in salps, 16.8% in krill), while krill tissue contained a higher PUFA proportion compared to salp tissue (13.8% in krill vs. 10.8% in salps).

**FIGURE 5:**
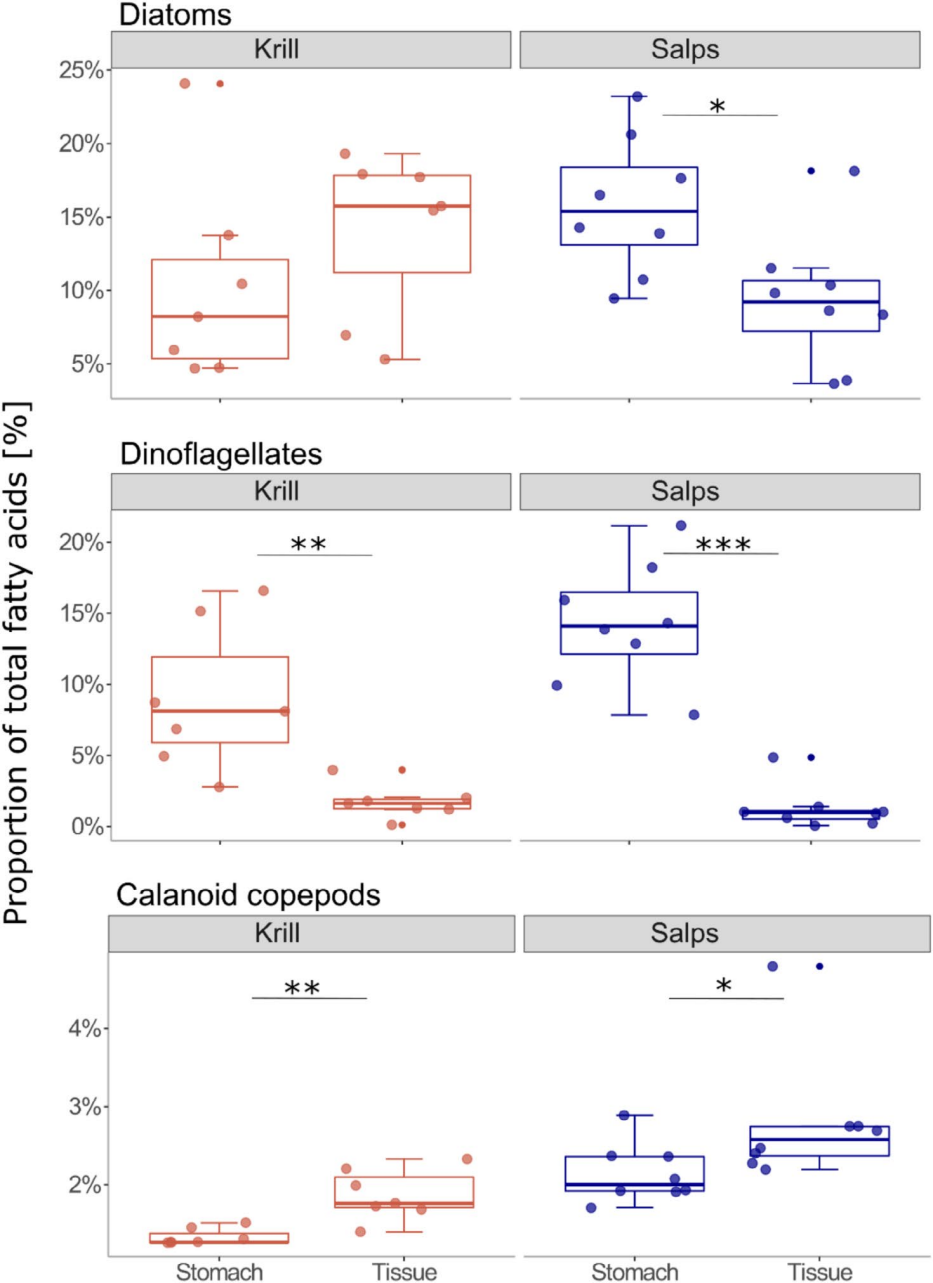
Fatty acid dietary markers from the tissue and stomach samples of krill and salps. Markers for diatoms (top; 16:1(*n*−7) & 20:5(*n*−3)), dinoflagellates (middle; 18:4(*n*−3) & 22:6(*n*−3)) and calanoid copepods (bottom; 20:1(*n*−11/*n*−9/*n*−7), 22:1(*n*−11/*n*−9/*n*−7)) are shown. Fatty acid markers are shown as a percentage of the total FA. Asterisks indicate the level of significance between stomach and tissue samples for each species, respectively (*p < 0.5, **p < 0.01, ***p < 0.001).

In salp stomachs, traditional diatom and dinoflagellate FA markers accounted for 15.8% and 14.3% of the total FA, respectively (Figure 5). These proportions were significantly higher compared to salp tissue (diatoms: *p* = 0.014, *t*‐test; dinoflagellates: *p* < 0.001, Wilcox test; Table S4). Alternative diatom and dinoflagellate FA markers represented 2.8% and 3.7%, respectively (Figure S7), with no significant difference between stomach and tissue samples (diatoms: 2.9%, dinoflagellates: 2.6%; Table S4). In contrast, FA markers for calanoid copepods were significantly higher in salp tissue compared to stomach samples (2.7% vs. 1.7%, *p* = 0.031, Wilcox test; Table S4).

For krill, traditional diatom markers were higher in tissue than in stomach samples (14.1% vs. 10.3%), although with considerable variability between samples (Figure 5). Alternative FA markers for diatoms were also significantly higher in krill tissue (tissue: 4.3%, stomachs: 2.16%; *p* = 0.035, *t*‐test; Table S4; Figure S7). Conversely, dinoflagellate markers were significantly higher in krill stomachs compared to tissues (9.0% vs. 1.7%, *p* = 0.004, Wilcox test, Table S4), while alternative dinoflagellate markers showed no significant difference (tissue: 3.18%, stomach: 2.06%). FA markers for calanoid copepods were significantly higher in krill tissue (1.3%) compared to stomach samples (0.1%, *p* = 0.005; Wilcox test).

Log‐ratio analysis of the observed differences in diatom and dinoflagellate markers between stomach and tissue samples revealed that traditional FA markers 16:1(*n*−7)/20:5(*n*−3) and 18:4(*n*−3)/22:6(*n*−3) contributed most to the explained variance in the first two PCA dimensions for both species (42.3% and 21.1% for krill; 35.6% and 24.9% for salps, respectively; Figure S8). Additionally, FA 18:1(*n*−9/*n*−7), indicative of detrital and carnivorous feeding (Phleger et al. 2002), also contributed to the explained variance.

## 4 Discussion

In this study, we addressed four critical methodological aspects to guide future research on zooplankton diet across both short and long time scales by comparing different processing methods and molecular markers. Our results show that sample processing and the use of different molecular markers, here 18S regions V4 and V7, significantly affect the detected prey community. Our findings highlight the importance of carefully choosing the marker region, as well as considering sample processing procedures before conducting molecular diet analysis. This is particularly important for species with highly efficient digestive enzymes, such as krill. Moreover, we show the synergistic effects of molecular methods with fatty acid analysis, providing insights into short‐ and long‐term dietary habits.

### 4.1 Comparison of 18S Variable Regions V4 and V7 for Krill Stomach Content

The prey community composition detected in the stomach contents of Antarctic krill varied notably between 18S variable regions V4 and V7. Libraries using region V7 contained a high number of parasite and predator sequences, which were removed to allow for a better comparison to the V4 libraries (Table S1). After removal, the filtered datasets revealed significant differences in prey composition between the two variable regions (Figure 1b). One example was the abundance of copepods, which were abundant in the V4 libraries (20%), but absent from V7 the libraries, despite the V7 primers matching the sequences of the two most abundant copepod taxa detected in the V4 libraries (ASVs identical to GenBank IDs KR048725.1, and KU064796.1) and successfully amplifying copepods in other studies (Cleary et al. 2012). Conversely, the V4 primers matched the sequence of one abundant flagellate group (Filosa‐Imbricatea) in the V7 libraries, which was not found in V4 libraries (ASV identical to GenBank ID FJ790725.1). These prey groups differ markedly in their morphology (large, hard bodied copepods vs. small soft‐bodied flagellates), which would be predicted to have very different digestion rates. Digestion biases may be particularly relevant when applying amplicons of different lengths, as longer amplicons (here V4) are lost to digestion more rapidly than shorter amplicons (Troedsson et al. 2009). This could explain the absence of more rapidly digestible flagellates in the V4 dataset. An additional potential bias in the diet composition could be related to the time‐of‐day krill were caught (see Discussion in Appendix S1).

Overall, our results comparing the variable regions V4 and V7 demonstrated that prey communities detected using the 18S rRNA can vary significantly depending on the chosen hypervariable region. This variability indicates that different variable regions are not directly comparable for assessing species composition across studies, regions or seasons. To improve comparability, researchers should carefully consider their choice of barcode region, and a combinatory approach using multiple barcode regions in parallel might help bridge differences between studies.

Multi‐marker approaches have been shown to enhance taxonomic resolution in diet studies by increasing the number of detected taxa and mitigating issues such as predator and parasite DNA masking prey sequences (da Silva et al. 2019; van der Loos and Nijland 2021). A potential combination could include the 18S rRNA gene alongside mitochondrial cytochrome oxidase subunit I (COI), which offers comparable taxonomic coverage (Giebner et al. 2020). COI is a suitable barcode for many metazoans (Bucklin et al. 2011), while 18S is well suited for phytoplankton (Lie et al. 2014; Tragin et al. 2018). This combination could be particularly valuable for diet studies of zooplankton with mixed, heterotrophic diets, such as krill and salps (Pakhomov et al. 2002). Further validation of the detected prey community could be realised by applying additional qPCR methods and/or microscopy for the identification of prey items (Frischer et al. 2014; Thompson et al. 2024). Moreover, it is of great importance to better understand primer biases, e.g., using mock communities (Parada et al. 2016) to establish validated primer which can be applied across studies.

#### 4.1.1 Application of Molecular Blocking Probes

In this study, we used blocking probes to reduce the amount of predator sequences in the analyses. However, the efficiency of these probes varied between the two variable regions. In the V4 libraries, predator DNA accounted for 20%–85% of sequences, with higher proportions likely stemming from individuals with an empty stomach, as observed in previous studies (Jungbluth et al. 2021). In contrast, V7 libraries consistently contained > 80% predator DNA, indicating poor blocking probe efficiency. This poor efficiency contrasts with the original study's results (Cleary et al. 2018), which applied the same blocking probe system and found that krill sequences comprised, on average, less than half of all reads, albeit with high variability between samples. One possible explanation is the use of a peptide nucleic acid (PNA) probe, which has poor water solubility (Shakeel et al. 2006), unlike regular DNA oligonucleotides. Consequently, incomplete solution might affect the efficiency of the blocking probe, which could be partially mitigated by additional steps (re‐suspension in dilute acid, heating) not included in this study. Additional potential biases are related to the two‐step PCR needed to amplify the V7 region due to the higher melting temperature of the PNA probe and the respective size selection during purification steps between PCRs. Moreover, if the efficiency of the blocking probe in the first PCR was low, the second PCR could cause a disproportionately high re‐amplification of the predator DNA. Despite multiple amplification and sequencing attempts to address potential methodological biases, we consistently observed poor blocking probe efficiency in the V7 libraries. The reason for the discrepancy between our results and those of Cleary et al. (2018) remains unclear.

#### 4.1.2 Parasites in the V7 Metabarcoding Libraries

The V7 libraries consisted of 75% reads from a eugregarine parasite (Cephaloidophoridae), a family including several species that commonly parasitise the digestive tract of Antarctic krill, other closely related krill species, and *Salpa thompsoni* (Takahashi et al. 2008; Wallis et al. 2017). Although Cephaloidophoridae can infect large proportions of krill populations and occur at high densities within their hosts, their ecological role remains poorly understood (Takahashi et al. 2011, 2008). The primers used for amplifying the V4 region did not match Cephaloidophoridae sequences, as confirmed by Nucleotide BLAST and Primer BLAST tools (Priyam et al. 2019), which could explain the disproportionate presence of this group in the V7 libraries. Other parasites, such as Syndiniales, fungi and nematodes, have also been identified in the digestive tract and faecal pellets of krill, salps, and copepods (this study; Cleary et al. 2019; Pauli et al. 2021; Zamora‐Terol et al. 2020), suggesting either host infection or the ingestion of infested prey, rather than direct consumption of parasites. From a methodological perspective, parasite sequences provide insights into additional, yet poorly understood trophic links. However, a high proportion of parasite sequences in diet‐focused studies might necessitate greater sequencing depth to accurately characterise ingested prey assemblages.

### 4.2 Impact of Sample Processing Prior to Preservation on Metabarcoding Library Composition

Evaluating how different processing treatments prior to preservation affect the composition of 18S V4 metabarcoding libraries of krill and salp stomachs, we observed significant differences between immediate and delayed freezing for krill stomach contents. The relative abundance of copepods, diatoms and parasitic dinoflagellates (Syndiniales) was approximately 30% higher in samples with delayed freezing compared to those that were immediately frozen. In contrast, salps, dinoflagellates and small flagellates (e.g., Ebriida) were more abundant in immediately frozen samples. We observed some variability between individual samples (Figure S3), while the overall trend remained consistent. The higher abundance of salps in immediately frozen samples aligns with their gelatinous nature, which makes them rapidly digestible and challenging to detect using traditional, visual methods for diet analyses. This has historically led to an underestimation of salp's contribution to their predators' diets (Henschke et al. 2016). Thus, the decline in salp sequences in krill stomachs with increasing processing time and delayed freezing is not surprising.

Our results further revealed differences in the digestion efficiency of diatoms, likely due to their silica shells, which remain intact for relatively long periods and can be difficult for some predators to digest (Gilmer and Harbison 1991; Passmore et al. 2006). Krill can efficiently crush diatom frustules using their gastric mill (Ullrich et al. 1991). However, the digestion of diatoms is slower compared to soft‐bodied prey. This may explain the higher abundance of diatom sequences in samples with delayed freezing. In contrast, the flagellate *Ebria* sp. (Filosa‐Thecofilosea), which has an internal silica skeleton (Hargraves 2002), was more abundant in immediately frozen samples. Internal skeletons such as these may hinder digestion less than the external skeletons of diatoms. Additionally, Ebriida are smaller (30–40 μm diameter) than most diatom taxa detected in our samples (e.g., *Chaetoceros*, *Thalassiosira* and *Fragilariopsis*) and their smaller size may facilitate more rapid digestion compared to larger or chain‐forming diatoms. Dinoflagellates (Dinophyceae) also showed slightly lower abundance in samples with delayed compared to immediate freezing, primarily driven by a decline in Peridiniales. This order includes both naked and thecate species, the latter possessing cellulose plates that may influence digestion efficiency.

Similarly, in salp stomachs, we observed differences in the digestion of flagellates and diatoms, both of which showed a higher abundance in samples with delayed freezing. This indicates that the digestion of tough body parts such as silica skeletons is slower in salps and supports the fatty acid analysis results presented in this study (see below). These findings are also consistent with previous work showing a high proportion of diatom sequences in salp faecal pellets, indicating low digestion efficiency (Pauli et al. 2021).

Overall, the differences between krill and salps in digestion are likely due to variations in digestive enzyme activity and the mechanical breakdown of prey. In krill, food processing involves high enzyme activity and efficient nutrient absorption, supported by mechanical grinding in the gastric mill. Recent transcriptome–proteome analyses of Antarctic krill revealed at least 14 nucleases targeting DNA and RNA, several of which are likely to be involved in digestion (Möller et al. 2022). Together with our results, this emphasises the importance of taking effective measures at an early stage to reduce DNA degradation when conducting molecular studies on krill, particularly in diet analyses. This combined effect of enzymatic and mechanical activity likely results in faster digestion of prey compared to salps (Saborowski 2012). In contrast, salp digestion efficiency depends on food quality and quantity, regional differences and salp size and developmental stage (von Harbou 2010). Furthermore, salp faecal pellets often contain significant amounts of undigested material, suggesting slower digestion rates compared to krill (Pauli et al. 2021; von Harbou 2010).

Our results highlight the importance of the time between sampling (catch) and preservation, particularly for species with highly efficient digestive enzymes, such as Antarctic krill. Delays in preservation can negatively affect the relative abundance of rapidly digested prey, such as salps and specific flagellate groups. Hitherto, freezing and ethanol are the most common preservation methods used in diet studies (Albaina et al. 2016; Cleary et al. 2018; Metfies et al. 2014). Both methods effectively preserve prey DNA if a freezing temperature of −80°C is used (Passmore et al. 2006; Weber and Lundgren 2009). However, freezing may lead to the degradation of prey DNA during dissection if samples are exposed to higher temperatures and begin to thaw (Passmore et al. 2006). In this study, we chose freezing to maintain comparability with simultaneously collected plankton samples. Frozen samples were dissected under a stereomicroscope equipped with a cooling system and supplemented with cold packs to prevent thawing. Therefore, we attribute the differences in prey community composition primarily to the extended processing time on board, which involved length measurements and staging of the animals before preservation. To minimise biases from dissection and sample pre‐processing, the use of DESS (salt saturated DMSO buffer with EDTA) for preservation could be a suitable alternative, as it has been shown to preserve high DNA quantity and quality while being easy to handle (van der Loos and Nijland 2021). Future studies could evaluate the use of EDTA/DESS for preservation directly after sampling to better understand whether this could be an appropriate method to help reduce nuclease activity in krill.

### 4.3 Separating Tissue and Digestive Tract for the Analysis of Fatty Acid Trophic Markers

Analyses of fatty acid (FA) trophic markers in zooplankton are often realised by pooling several individuals of the same species to obtain sufficient biomass (Auel et al. 2002; Hiltunen et al. 2015). However, using whole animals prevents the differentiation of short‐term FA signals from food ingested immediately before sampling and FA signals stored in tissues over longer time scales. While this distinction may not be the focus of every study, separating the digestive tract from the tissue could yield significantly different results. In this study, we compared the FA profiles in both stomach and tissue samples from the same krill and salp individuals to shed light on this. Our results revealed significant differences in the proportion of FA trophic markers for diatoms, dinoflagellates, and copepods in both species, highlighting the importance of separating stomach and tissue when analysing FA trophic markers to distinguish between short‐ and long‐term signals. Furthermore, comparing FA signals from both stomachs and tissue samples provides a more accurate assessment of the assimilation efficiency of specific prey groups. Thus, for studies aiming to differentiate between short‐ and long‐term FA signals, we recommend conducting separate analyses of tissue and digestive tract, even when pooling multiple individual animals. In this context, we emphasise the need for detailed reporting of sample processing procedures in the appropriate manuscript sections or [Link], [Link], [Link], [Link], [Link], [Link]. We found that information on whether the digestive tract was removed prior to FA analysis is often omitted (e.g., Reiss et al. 2015; Stübing et al. 2003; von Harbou et al. 2011), which can hinder the reproducibility and interpretation of results.

Supporting our metabarcoding results, we found significantly higher diatom markers in salp stomachs compared to their tissue, while the opposite trend was observed for krill. These findings also align with other studies on the digestion and assimilation efficiency of diatoms, suggesting that krill can assimilate diatoms more efficiently than salps (Ullrich et al. 1991; von Harbou et al. 2011). Salps lack the anatomic features to crush diatom frustules, a function performed in the gastric mill in krill (Foxton 1966; Ullrich et al. 1991). Similarly, a significantly higher proportion of dinoflagellate markers in stomach compared to tissue samples in both species indicates a low assimilation efficiency for dinoflagellates. However, it remains unclear whether and to what extent flagellate parasites, such as Syndiniales, found in both species' stomach contents using molecular methods (Pauli et al. 2021), are reflected by these trophic FA markers. Moreover, for dinoflagellates, we observed significantly different results between stomach and tissue for both krill and salps, applying different FA marker combinations. This underscores the importance of carefully selecting FA combinations, particularly when comparing short‐term gut signals with long‐term tissue signals.

In addition to trophic markers for specific groups, the analysis of whole versus dissected animals may also influence total lipid content. A recent study by Pauli et al. (2021) found significantly lower total lipid levels in dissected krill compared to whole animals collected in the same season. Alonzo et al. (2005) observed differences in the FA composition between the digestive tract and other body parts, showing that experimentally induced dietary changes were more prominently reflected in the lipid classes and FA composition of the digestive gland of krill, as opposed to whole‐body samples. Similarly, Schmidt et al. (2006) reported that the total lipid content in the digestive gland of krill was approximately 1.5‐times higher than in muscle tissue.

### 4.4 Combining Metabarcoding and Fatty Acid Analyses

In this study, we analysed the diet composition of krill and salps using 18S metabarcoding to target the short‐term diet in stomach contents and complement this with FA analysis from stomachs and tissue to capture both short‐ and long‐term dietary signals.

Metabarcoding of krill and salp stomachs using variable region V4 revealed a significant contribution of flagellates to the diet of both species, with a wide diversity of taxa, including Dinophyceae, parasitic Syndiniales, and several cercozoan flagellates (Filosa). Consistent with these findings, FA markers for dinoflagellate markers showed a high proportion in the stomachs of krill and salps, indicating that flagellates play a crucial role in their short‐term diet. In contrast, long‐term FA signals in the tissue were relatively low. Similarly, Metfies et al. (2014) supported the FA analysis from von Harbou et al. (2011) by using 18S metabarcoding of two salp species in the Lazarev Sea, revealing a flagellate‐based diet of salps with differences between co‐occurring species.

For calanoid copepods, FA markers (20:1/22:1) were significantly more abundant in the tissue of both krill and salps compared to their stomachs, suggesting either a high assimilation efficiency of copepods or that copepods were consumed earlier. This finding supports the metabarcoding results for krill and completes the data obtained for salps. Here, we observed a higher proportion of copepod sequences in immediately frozen samples, indicating the negative effect of the prolonged processing time in samples with delayed freezing. Moreover, long‐term FA markers in the tissue of salps suggest a consistent contribution of calanoid copepods to their diet, further supporting our metabarcoding results and underscoring the importance of combining short‐ and long‐term dietary markers.

FA markers are generally restricted to a coarse taxonomic resolution, while metabarcoding provides a finer resolution, potentially down to species level. In this study, the combined metabarcoding‐FA approach enabled a more detailed analysis of various flagellate groups, including the identification of parasitic flagellates (Syndiniales) as regular components in krill and salp stomachs. The development of specific trophic markers to detect parasitic flagellates could help to further enhance our understanding of parasite–host interactions. Additionally, studies from other fields have highlighted the value of integrating metabarcoding with quantitative FA analyses. For example, analyses of polar bear faeces using this combined approach revealed additional prey species (Franz et al. 2023). Similarly, Lewe et al. (2021) demonstrated that employing a combination of 16S metabarcoding and phospholipid fatty acid (PLFA) analyses to investigate microbial communities in both terrestrial and aquatic soils allowed PLFAs to function as a proxy for biomass, thereby refining the relative abundances derived from metabarcoding.

Overall, these results demonstrate that combining metabarcoding and FA analyses is a powerful approach for refining zooplankton diet studies. This integrated method provides valuable insights that might otherwise be overlooked, especially when differentiating between short‐ and long‐term dietary signals, thereby enhancing the interpretation of trophic interactions.

## Author Contributions

N.‐C.P., K.M., M.H.I. and B.M. developed research ideas and conceived the study design. N.‐C.P., B.M. and M.H.I. collected samples and performed fieldwork. N.‐C.P. and K.M. conceptualised and designed the molecular analyses. M.G. provided analytical tools for fatty acid analyses. A.C.C. provided V7 data. N.‐C.P. performed laboratory work and conducted analyses with the help of S.N., K.M. and M.G. N.‐C.P., K.M. and B.M. conceptualised the manuscript; N.‐C.P. wrote the manuscript; and all authors contributed to manuscript revisions.

## Disclosure

Benefit sharing: All samples were collected in compliance with the terms and regulations of the Antarctic Treaty. A permit was granted by the German Environment Agency, reference number II 2.8‐94003‐3/409. Benefits from this work emerge from sharing data and results on public databases as described above.

## Conflicts of Interest

The authors declare no conflicts of interest.

## Supporting information


**Appendix S1:** men70030‐sup‐0001‐AppendixS1.pdf.

**Appendix S2:** men70030‐sup‐0002‐AppendixS2.csv.

**Appendix S3:** men70030‐sup‐0003‐AppendixS3.csv.

**Appendix S4:** men70030‐sup‐0004‐AppendixS4.csv.

**Appendix S5:** men70030‐sup‐0005‐AppendixS5.csv.

**Appendix S6:** men70030‐sup‐0006‐AppendixS6.csv.

**Appendix S7:** men70030‐sup‐0007‐AppendixS7.csv.

## Data Availability

All raw, paired‐end sequence reads are available in the European Nucleotide Archive (ENA), under the accession number PRJEB43900 (https://www.ebi.ac.uk/ena/browser/view/PRJEB43900). Metadata and additional tables and figures are available in the [Link], [Link], [Link], [Link], [Link], [Link] of this manuscript. Code and R scripts were deposited in the GitHub repository ncpauli/RefiningDietStudies and are available via Zenodo, doi 10.5281/zenodo.14289105. All samples were collected in compliance with the terms and regulations of the Antarctic Treaty. A permit was granted by the German Environment Agency (reference number II 2.8‐94003‐3/409).
