# Supplementary material for: Refining Zooplankton Diet Composition Studies Over Short and Long Time Scales by Combining 18S Metabarcoding With Fatty Acid Analyses
Source: Mol Ecol Resour. 2025 Aug 18;25(8):e70030. doi: 10.1111/1755-0998.70030 (PMC12550472; doi:10.1111/1755-0998.70030)
Supplement: Supplementary file 1 — Appendix S1: men70030‐sup‐0001‐AppendixS1.pdf. [file MEN-25-e70030-s002.pdf]

MOLECULAR ECOLOGY

Supplemental Information for:

Refining zooplankton diet composition studies over short- and long-time scales by combining 18S metabarcoding with fatty acid analyses

Nora-Charlotte Pauli, Katja Metfies, Stefan Neuhaus, Martin Graeve,
Alison C. Cleary, Morten H. Iversen & Bettina Meyer

Table of Contents:

**Material & Methods**	Page 2
**Discussion**	Page 2
**Supplemental Tables**	Page 3
**Supplemental Figures**	Page 7
**References**	Page 16

MOLECULAR ECOLOGY

Supplemental Material and Methods

Sequencing analysis pipeline

The 3'-ends of the reads were trimmed at the first appearance of an ambiguous base (N). Subsequently, an external call of the tool *cutadapt* , v.2.10 (Martin, 2011) removed the primer segments from the 5'-ends. As the amplified V7 region is shorter than the read length, primer segments also occur at the 3'-ends of the corresponding reads and were removed as well. Base qualities of the reads usually drop towards the 3'-ends and reads need to be truncated accordingly. Based on the visual examination of the run-specific quality plots of the forward and reverse reads, 3'-truncation lengths for both reads were specified for each run individually. Thus, forward reads of the V4 runs were truncated at base position 270 and reverse reads of the V4 runs were truncated between base positions 220 and 260. The V7 reads were trimmed to a shorter length of 210 bp for the forward and 160 bp for the reverse reads. Additionally, reads were filtered based on the number of expected errors per sequence, which were calculated from the base quality scores. The maximum number of expected errors per sequences allowed was derived from the lengths of the truncated reads by dividing it by 100 (i.e., 2.7 and 2.2–2.6 for V4 reads and 2.1, 1.6 for V7 reads). Subsequently, parametric error models, which model the base errors introduced during amplicon sequencing, were calculated for each read direction and each sequencing run separately. These error models were used to correct amplicon errors by the DADA2 algorithm (Callahan et al., 2016). Denoised reads were merged with a required minimum read overlap of 20 bp, disallowing any mismatches. These denoised amplicons are termed Amplicon Sequence Variants (ASV). ASV abundance tables were constructed and merged per variable region. Subsequently, a chimera-detection method was applied and predicted chimeric sequences were removed from the tables. Sample-demultiplexing was performed by the MiSeq-reporter-sequencing software.

Supplemental Discussion

All krill individuals used to compare V4 and V7 were collected at night, except for three individuals captured in the Bransfield Strait (Table S1). Krill typically feed at night when they migrate to the upper 200 m (Tarling & Fielding, 2016). Thus, different sampling times could introduce biases in diet composition analyses. However, in this study, the respective krill stomachs (K1101, K1109, K1126; Figure S9) exhibited similar diet composition patterns as the remaining krill caught during at night (e.g., dominance of parasites, absence of copepods).

MOLECULAR ECOLOGY

Supplemental Tables

Table S1. Detailed sampling overview of all krill and salps *used for metabarcoding of the 18S rDNA variable regions V4 and V7, as well as for fatty acid analysis of stomach and tissue. *☼ Day was defined as the period from 06:00 a.m. to 19:00 p.m. (UTC – 03:00). ☽ Night was defined as the period from 19:00 p.m. to 06:00 a.m. according to the local times of sunrise and sunset.*

**Region and species**	**Freezing**	**Date**	**Sample size (n)**	**Sampling device**	**UCT Day/Night***	**Latitude/ Longitude**
**Comparing molecular markers V4 *vs.* V7**						
**Krill**						
Deception Island	Delayed	30.03.2018	V4 n = 3<br>V7 n = 3	IKMT	03:41 ☽	-62.966147/<br>-60.465931
Elephant Island	Delayed	08.04.2018	V4 n = 3<br>V7 = 3	IKMT	23:25 ☽	-60.9971/<br>-54.836851
South Shetland Islands	Delayed	05./06.04.2018	V4 n= 5<br>V7 n = 6	IKMT	00:04 ☽<br>02:24 ☽	-61.742393/<br>-59.018374
Weddell Sea	Delayed	19.04.2018	V4 n= 3<br>V7 n = 3	IKMT	07:44 ☽	-62.740251/<br>-56.505496
Bransfield Strait West	Delayed	26./27.03.2018	V4 n = 5<br>V7 n= 6	IKMT	06:15 ☽<br>10:22 ☼	-62.583684/<br>-59.702034
**Comparing treatments immediately *vs.* delayed freezing**						
**Krill V4**						
Weddell Sea	Delayed	19.04.2018	10	IKMT	07:35 ☽	-62.740251-<br>56.505496
	Immediate	13.04.2018	10	RMT	15:47 ☼	-62.607847<br>-54.572816
**Salps V4**						
Deception Island	Delayed	30.03.2018	10	IKMT	03:41 ☽	-62.966147<br>-60.465931
	Immediate	31.03.2018	10	IKMT	08:34 ☽	-62.999575<br>-60.459676
**Fatty acids**						
Bransfield Strait West	Delayed	26.03.2018<br>27.03.2018<br>28.03.2018	Krill N = 2<br>Salps N = 5	IKMT	10:13 ☼<br>06:06 ☽<br>05:54 ☽	-62.536-62.587/<br>-59.404-59.926
Deception Island	Delayed	30.03.2018	Krill N = 1<br>Salps N = 3	IKMT	03:41 ☽	-62.966147/<br>-60.465931
Elephant Island	Delayed	08.04.2018	Krill N = 1<br>Salps N = 2	RMT	23:07 ☽	-61.008494/<br>-54.840557
South Shetland Islands	Delayed	04.04.2018<br>05.04.2018<br>05.04.2018	Krill N = 2<br>Salps N = 3	IKMT	23:01 ☽<br>23:56 ☽<br>02:24 ☽	-61.503-61.745/<br>-59.024-60.527

MOLECULAR ECOLOGY

Weddell Sea	Delayed	19.04.2018	Krill N = 1<br>Salps N = 1	IKMT	07:35 ☽	-62.746706/<br>-56.502802
Bransfield Strait East	Delayed	20.04.2018	Krill N/A<br>Salps = 3	IKMT	16:03 ☼	-61.888996/<br>-53.920532

Table S2. Results of the permutational multivariate analysis of variance (PERMANOVA). *For krill stomachs, two different molecular markers (18S variable regions V4 and V7) were compared. For stomachs of krill and salps, two different pre-processing procedures were compared (Immediate vs. delayed freezing). PERMANOVA was performed using 9999 permutations and Euclidean distances. Significant results are highlighted in bold.*

**Analysis**	**Df**	**Sums of Squares**	**F value**	**p-value**
Comparing molecular markers				
**Krill V4 vs. V7** (with parasite)	1	1820.0	7.814	**<0.001**
**Krill V4 vs. V7** (no parasite)	1	363.3	3.678	**<0.001**
Comparing freezing treatments				
**Krill V4** Immediate vs. delayed	1	2452.0	1.2618	**0.009**
**Salps V4** Immediate vs. delayed	1	625.7	1.376	0.128

MOLECULAR ECOLOGY

Table S3. Mean proportions of the single fatty acids extracted from stomach and tissue samples from krill (Euphausia superba) and salps (Salpa thompsoni). *Single fatty acids are shown as percent of the total fatty acids. The average share of the single identified fatty acids is shown for each species, and sample type (stomach vs. tissue), respectively.*

	Euphausia superba		*Salpa thompsoni*	
Fatty acid	Stomach	Tissue	Stomach	Tissue
14:0	3.07	4.56	1.02	3.55
14:1(n-5)	0.04	0.08	0.10	0.05
15:0	0.15	0.21	0.60	1.65
16:0	10.17	13.54	6.84	13.26
16:1(n-7)	1.85	3.05	1.64	2.74
16:1(n-5)	0.12	0.21	0.45	0.80
16:2(n-4)	0.06	0.33	0.13	0.02
16:3(n-4)	0.11	0.26	0.16	0.12
16:4(n-1)	0.15	0.70	0.84	0.11
18:0	3.70	0.65	1.46	3.31
18:1(n-9)	5.74	3.23	1.11	3.65
18:1(n-7)	2.52	4.49	2.24	2.27
18:1(n-5)	0.07	0.11	0.12	0.18
18:2(n-6)	0.59	1.11	0.76	1.10
18:3(n-3)	0.41	0.50	0.32	0.21
18:4(n-3)	1.06	1.57	2.60	1.25
20:1(n-11)	0.04	0.07	0.38	0.21
20:1(n-9)	0.05	0.36	0.15	0.41
20:1(n-7)	0.17	0.12	0.52	1.18
20:2(n-3)	0.06	0.26	0.51	0.01
20:2(n-6)	0.07	0.19	0.09	0.20
20:3(n-3)	0.14	0.10	0.37	0.21
20:4(n-3)	0.11	0.22	0.13	0.08
20:5(n-3)	8.42	11.01	14.15	6.55
22:1(n-11)	0.04	0.20	0.13	0.78
22:1(n-9)	0.11	0.25	0.19	0.21
22:1(n-7)	0.05	0.12	0.21	0.10
22:2(n-6)	0.12	0.05	0.81	0.02
22:5(n-3)	7.97	0.15	11.67	0.02
22:6(n-3)	0.05	5.08	0.08	5.98

MOLECULAR ECOLOGY

Table S4. Results of the t-test and Wilcoxon rank sum test comparing fatty acid markers. *Differences in the means of the dietary fatty acid markers for diatoms, dinoflagellates, and calanoid copepods between tissue and stomach content samples in krill and salps were tested. Significant results are highlighted in bold and asterisks depict the level of significance (* <0.05, ** <0.01, *** <0.001).*

**T-test**	**Df**	**T-statistic**	**p-value**
Diatoms (traditional)			
Krill	12	-1.128	0.2812
Salps	14	2.802	**0.0141***
Diatoms (alternative)			
Krill	12	-2.362	**0.0359***
Salps	14	-0.237	0.8079
**Wilcoxon rank sum test**		**W**	**p-value**
Dinoflagellates (traditional)			
Krill		48	**0.004****
Salps		64	**<0.001*****
Dinoflagellates (alternative)			
Krill		10	0.0736
Salps		48	0.1036
Copepods			
Krill		2	**0.005****
Salps		11	**0.031***

MOLECULAR ECOLOGY

Supplemental Figures

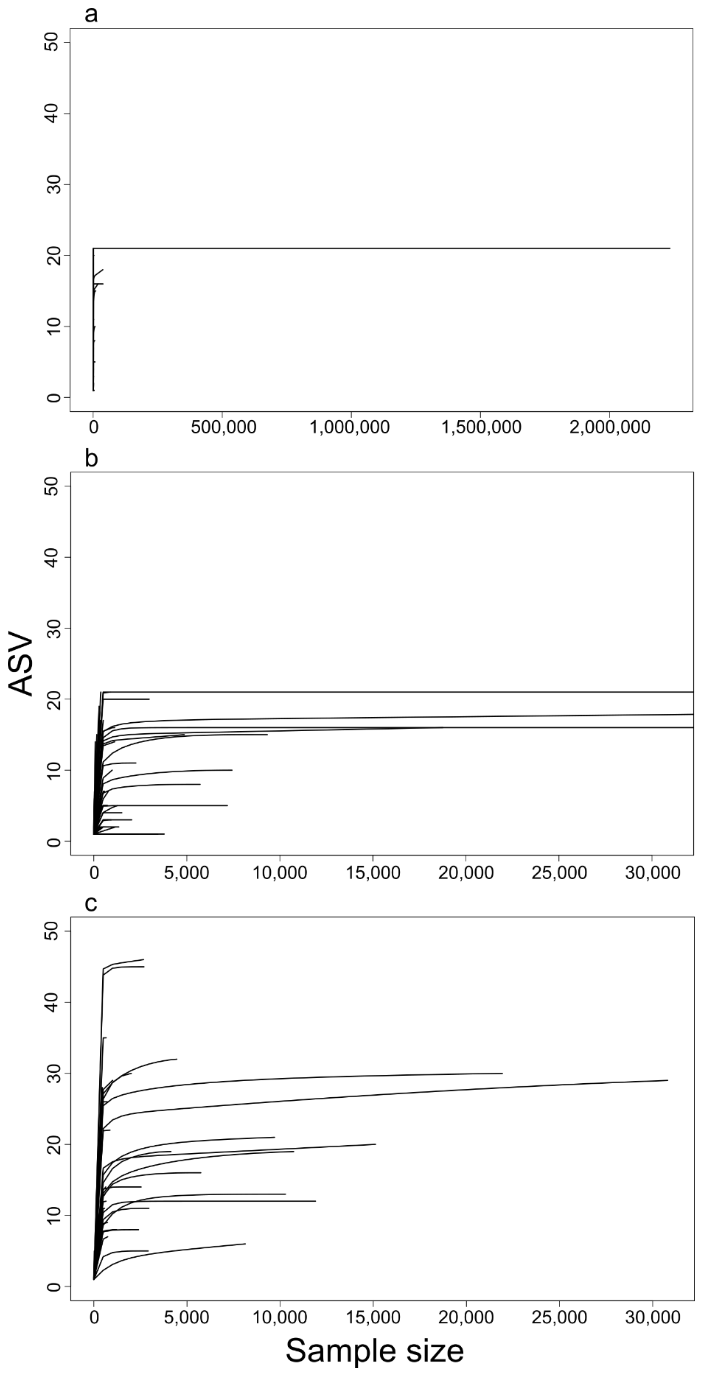

Figure S1. Rarefaction curves of the sequencing libraries of krill stomach samples comparing 18S variable regions V4 and V7. *Both variable regions are shown together. a) Rarefaction curve of the raw dataset including predator (i.e., krill) sequences. b) Same data set as in a) with a zoom on the x-axis to allow a better comparison to panel c. c) Rarefaction curves after the removal of predator sequences. Note the different x-axis scales of a to b and c.*

MOLECULAR ECOLOGY

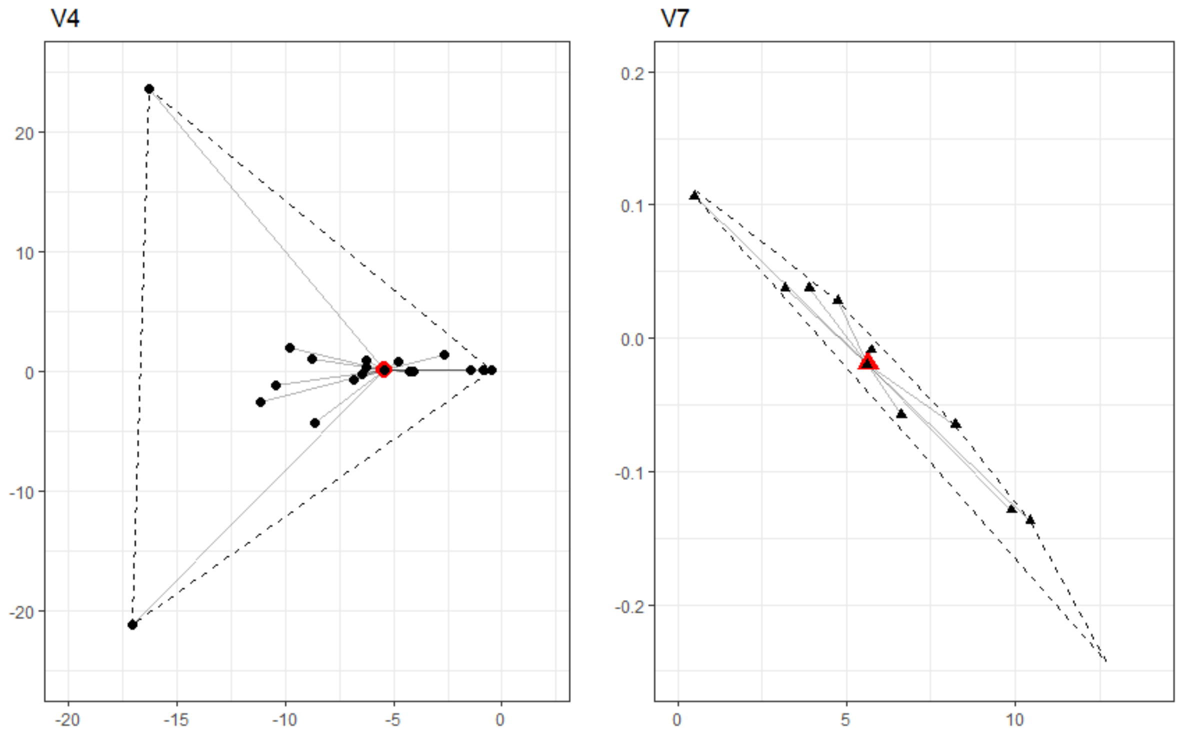

Figure S2. Group dispersion of the sequencing libraries of krill stomach samples using two different variable regions of the 18S *rDNA, variable region V4 (left) and region V7 (right). Group dispersion was tested using the betadisper function in the vegan package in R (Oksanen et al. 2019). Visualization was conducted following a script by https://chrischizinski.github.io/rstats/adonis/.*

MOLECULAR ECOLOGY

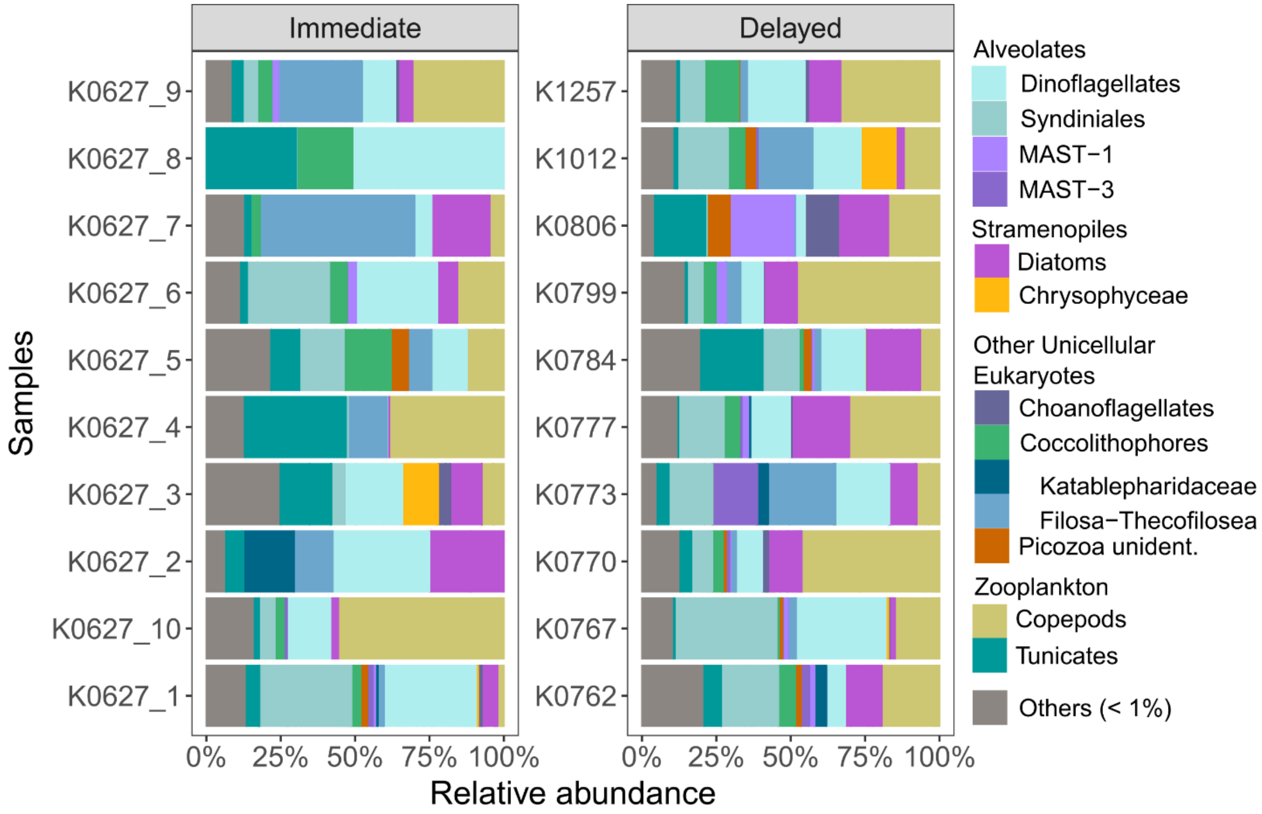

Figure S3. Krill stomach content using 18S variable region V4. *Relative abundance of taxa on the taxonomic level of Class in the stomach content of krill comparing two different pre-processing procedures. Samples that were immediately frozen in bulk at -80 °C after the catch was on board (left panel) and samples with delayed freezing (right panel) due to measuring and staging of krill prior to freezing in separate bags at -80 °C. Taxa with a relative abundance of <1% were grouped as 'Others'.*

MOLECULAR ECOLOGY

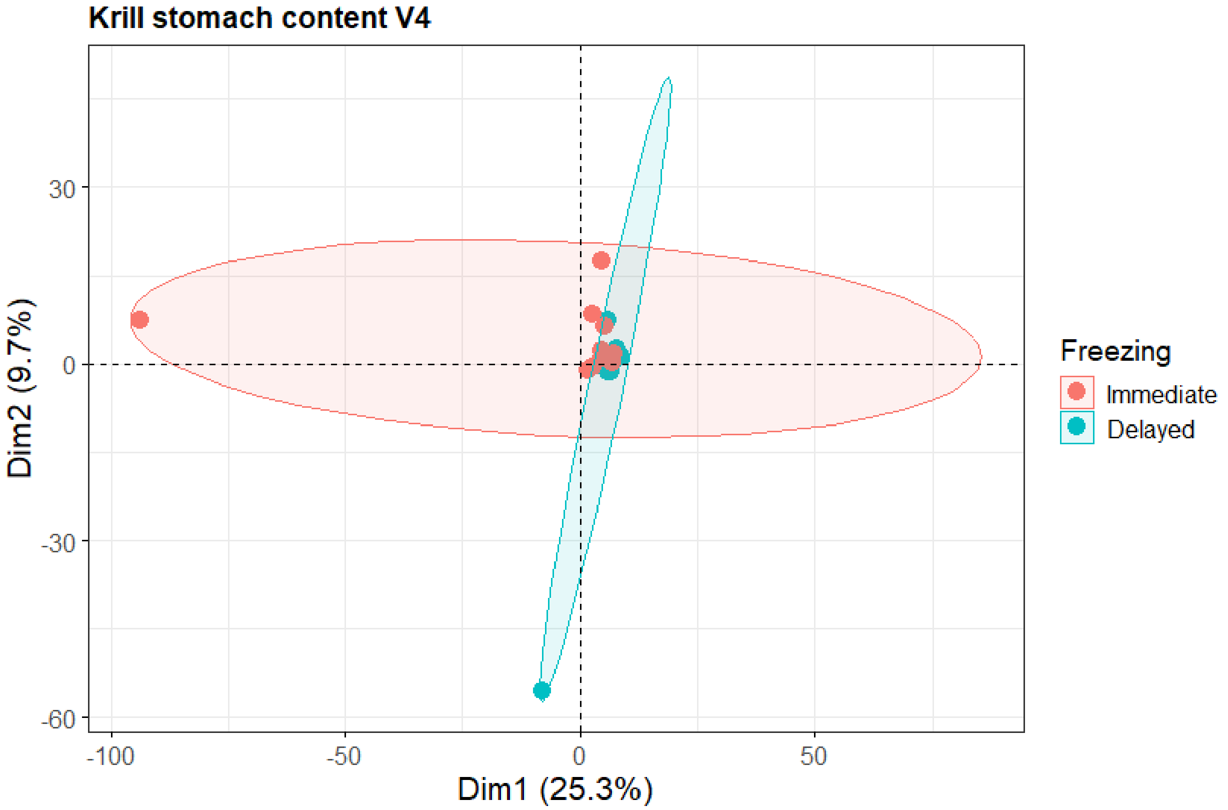

Figure S4. Principal component analysis (PCA) of krill stomach content using variable region V4. *PCA of the V4 sequencing libraries of krill stomachs using Euclidean distances for the centre-log ratio transformed data comparing two treatments: immediately frozen samples (red) and samples with delayed freezing (blue). The ellipses represent a 95% confidence interval for each treatment group. The explained variance by the first and second dimension are shown in percent on the x- and y-axis, respectively.*

MOLECULAR ECOLOGY

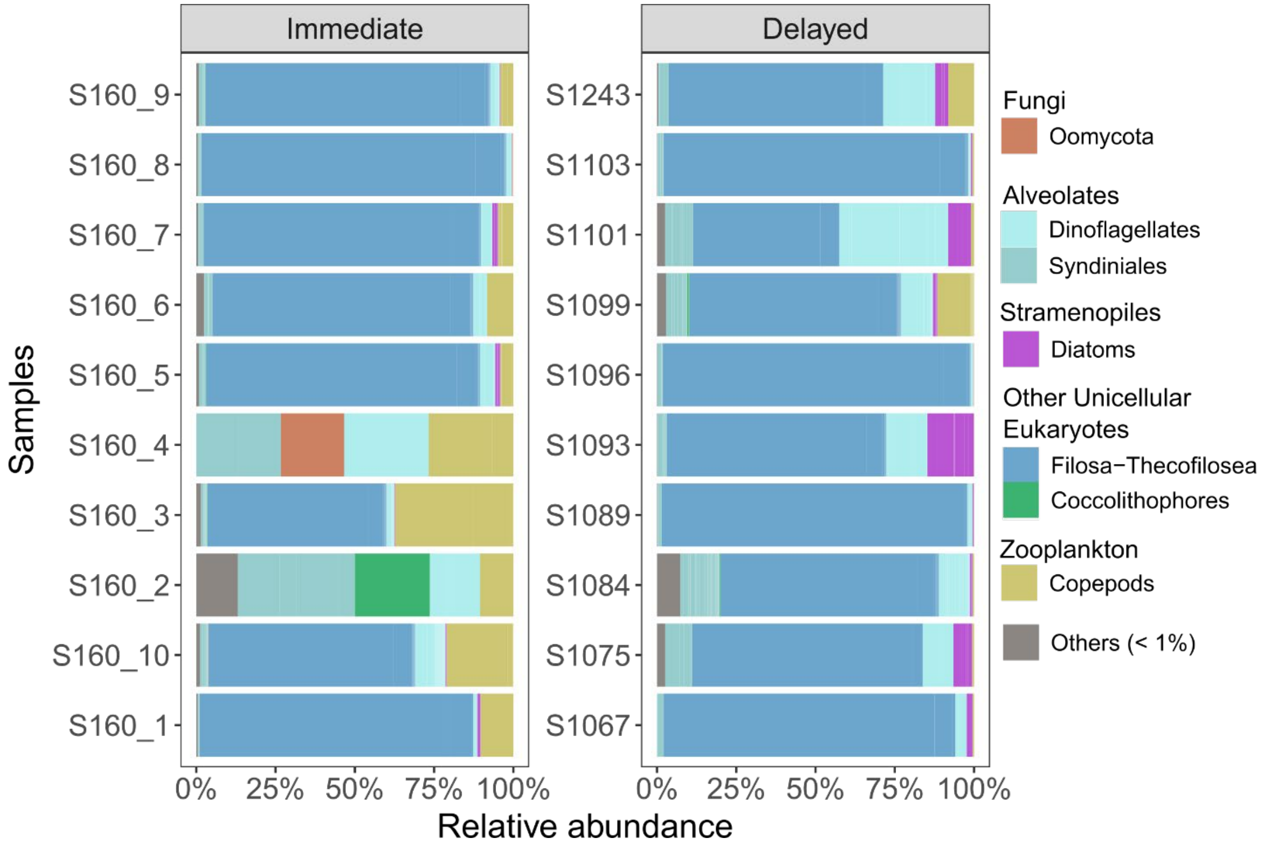

Figure S5. ***Relative abundance of taxa in salp stomachs using 18S variable region V4****. Taxa on the level of Class found in the V4 metabarcoding libraries of salp stomach content samples, comparing two treatments: in the left panel, samples were frozen in bulk at -80 °C immediately after catch. In the right panel, samples were frozen with a delay due to measurements and staging of salps. Taxa with a relative abundance of less than 1% were grouped as "Others".*

MOLECULAR ECOLOGY

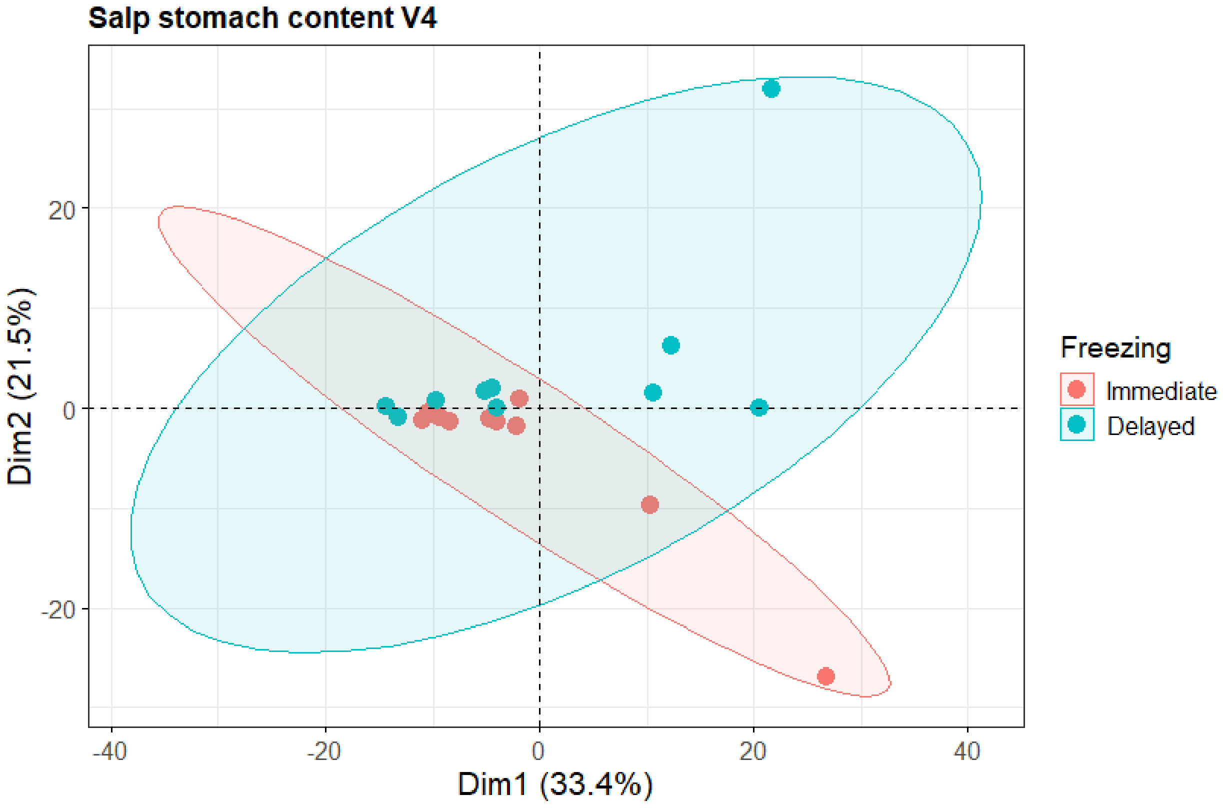

Figure S6. Principal component analysis (PCA) of salp stomach content using 18S variable region V4*. PCA of the V4 sequencing libraries of salp stomachs using Euclidean distances for the centre-log ratio transformed data comparing two treatments: immediately frozen samples (red) and samples with delayed freezing (blue). The ellipses represent a 95% confidence interval for each treatment. The explained variance by the first and second dimension are shown in percent on the x- and y-axis, respectively.*

MOLECULAR ECOLOGY

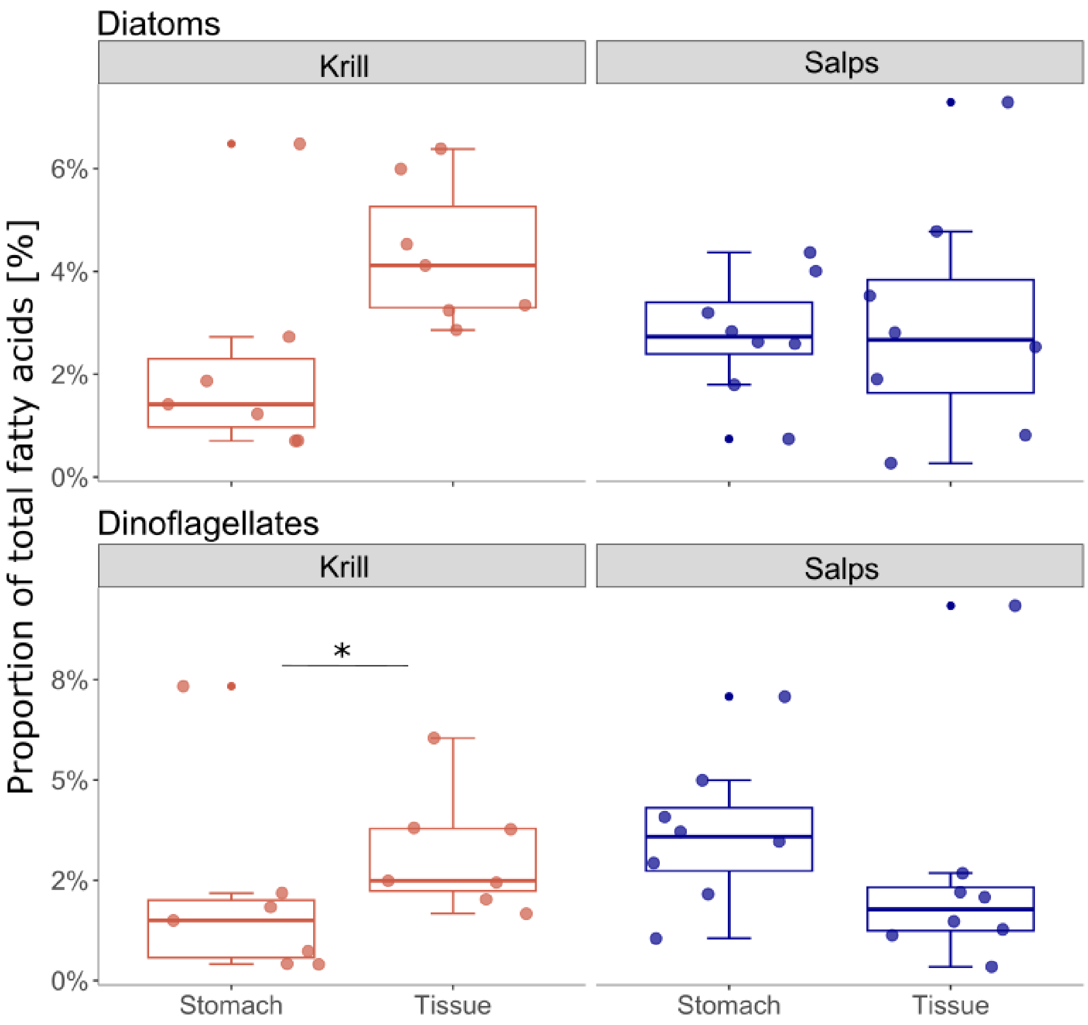

Figure S7. Alternative fatty acid dietary markers from the tissue and stomach samples of krill and salps *omitting membrane fatty acids 20:5(n-3) and 22.6(n-3). Markers for diatoms (16:1(n-7) and all identified C16 PUFAs (16:2(n-4), 16:3(n-4), 16:4(n-1)), as well as dinoflagellates (18:4(n-3) and all C18 PUFAs (18:3(n-3), 18:2(n-3)) are shown, respectively. Fatty acid markers are shown as percentage of the total fatty acids. Asterisks indicate the level of significance between stomach and tissue samples for each species, respectively (* p <0.5).*

MOLECULAR ECOLOGY

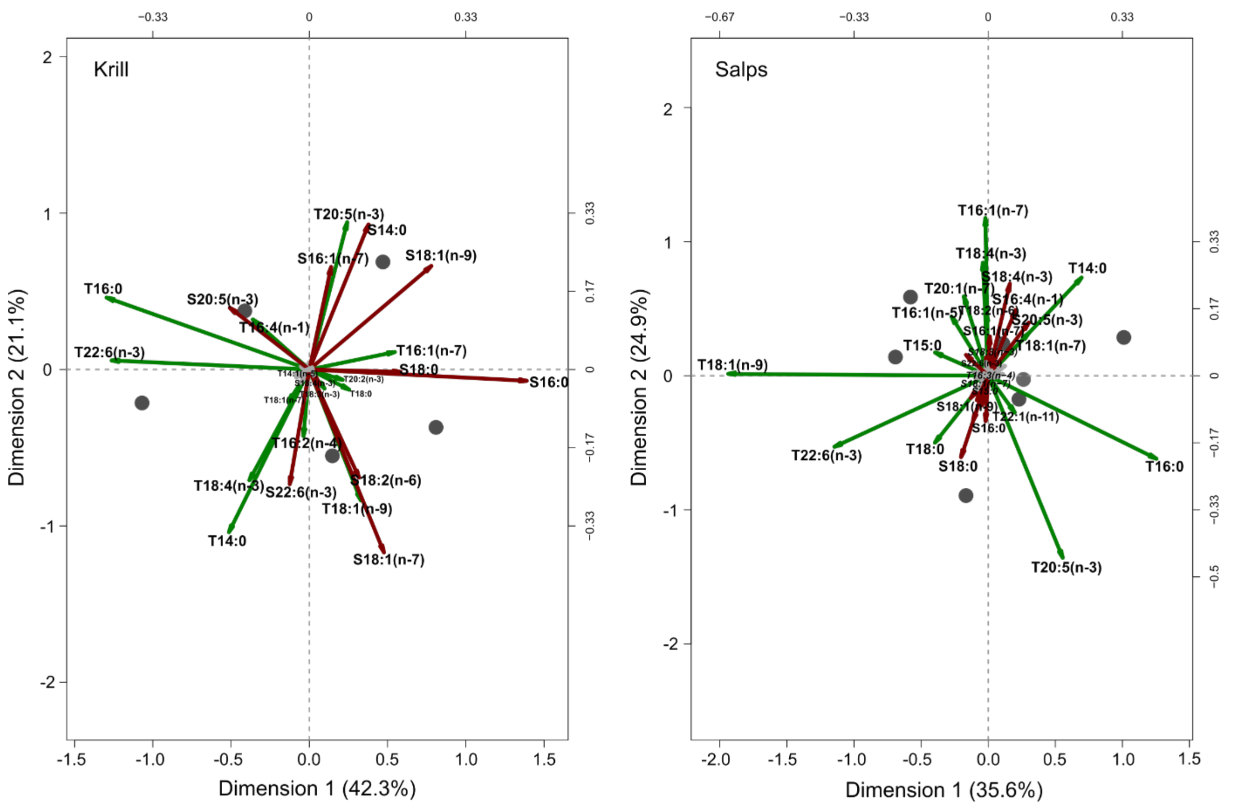

Figure S8. Biplot of the log-ratio analysis of the fatty acid composition in the tissue and stomach of krill (left) and salps (right). *The explained variance by the first two dimensions is shown in percent. Fatty acids extracted from the tissue are depicted by a capital T in green, fatty acids extracted from the stomach content are depicted by a capital S in red. The length of the arrows corresponds to the relative contribution of the single fatty acids to the explained variance, with longer arrows representing a higher contribution. Labels of fatty acids with a very low contribution in the centre of the plot were removed for clarity.*

MOLECULAR ECOLOGY

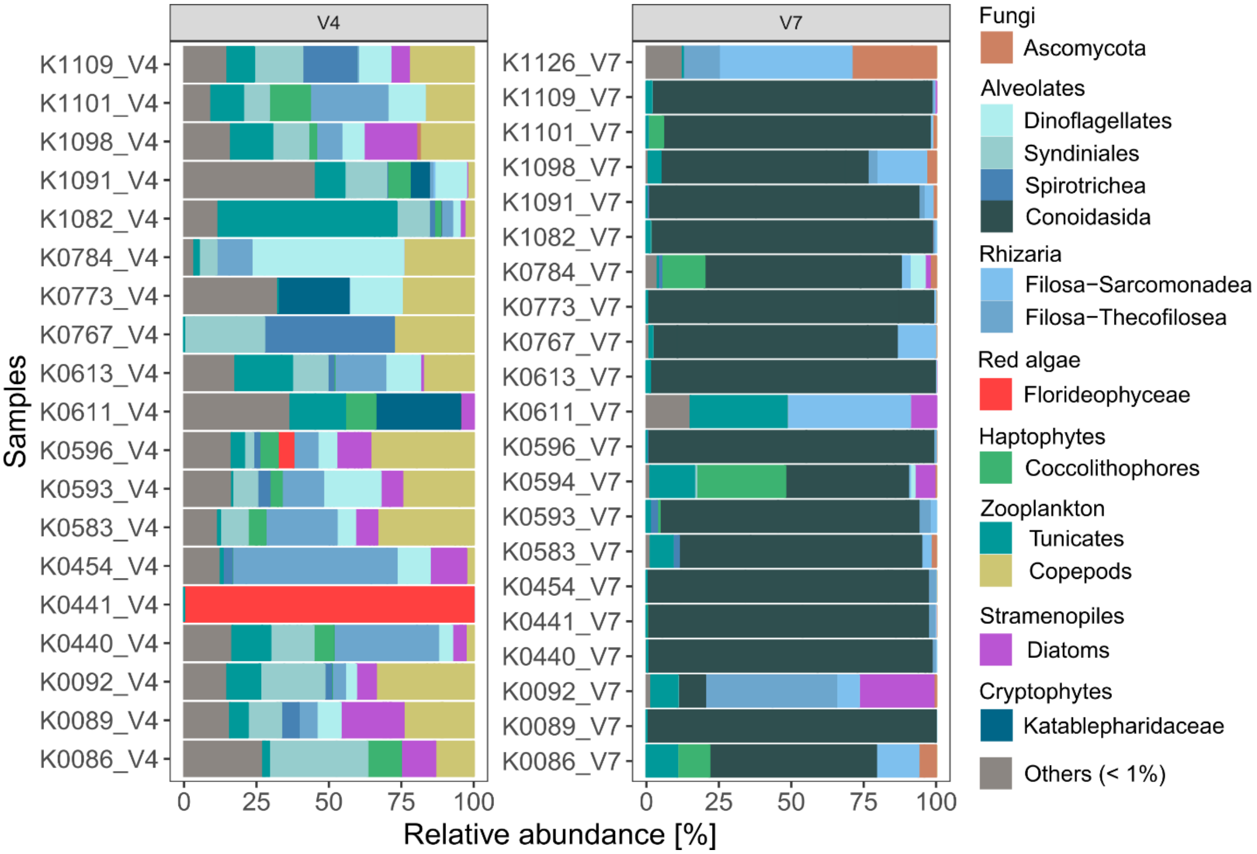

*Figure S9. **Relative abundance of taxa in krill stomachs for individual samples**. Taxa on the level of Class found in the V4 (left) and V7 (right) metabarcoding libraries of krill stomach content samples. Taxa with a relative abundance of less than 1% were grouped as "Others".*

References

Callahan, B. J., McMurdie, P. J., Rosen, M. J., Han, A. W., Johnson, A. J. A., & Holmes, S. P. (2016). DADA2: High-resolution sample inference from Illumina amplicon data. *Nature Methods*, *13*(7), p. 581-583. https://doi.org/10.1038/nmeth.3869

Martin, M. (2011). Cutadapt removes adapter sequences from high-throughput sequencing reads. *EMBnet.journal Bioinformatics in Action*, *17*(1). https://doi.org/10.14806/ej.17.1.200

Tarling, G. A., & Fielding, S. (2016). Swarming and Behaviour in Antarctic Krill. In V. Siegel (Ed.), *Biology and Ecology of Antarctic Krill* (p. 279-319). Springer International Publishing. https://doi.org/10.1007/978-3-319-29279-3_8
